# Enhancing optical properties and stability of DNA-functionalized carbon nanotubes with cryoprotectant-mediated lyophilization

**DOI:** 10.1016/j.carbon.2025.121159

**Published:** 2025-12-24

**Authors:** Aceer Nadeem, Aidan Kindopp, Ella Junge, Maryam Rahmani, Daniel Roxbury

**Affiliations:** a Department of Chemical Engineering, University of Rhode Island, Kingston, RI, 02881, United States; b School of Chemistry and Biochemistry, Georgia Institute of Technology, Atlanta, GA, 30322, United States

**Keywords:** Single walled carbon nanotubes, Lyophilization, Cryoprotectants, Sensor stability, Long-term storage, Near infrared fluorescence

## Abstract

The long-term optical performance and stability of single-walled carbon nanotubes (SWCNTs) functionalized with single-stranded DNA are critical for their application in near-infrared (NIR) fluorescence biological sensing and imaging. However, the aggregation of such DNA-SWCNTs during storage presents a significant challenge. Here, we explored the use of lyophilization combined with various cryoprotectants to enhance the long-term stability and reconstitution of DNA-SWCNTs at room temperature. Five conventionally used cryoprotectants, including glucose, sucrose, mannitol, polyethylene glycol (PEG), and polyvinyl alcohol (PVA), were evaluated for their ability to maintain desired optical properties and prevent aggregation of SWCNTs through the process of lyophilization and reconstitution. Our results indicated that glucose and PEG, particularly in an 80:20 ratio by weight, provided the best performance, preserving NIR fluorescence and ensuring consistent reconstitution without significant aggregation. Further, *in vitro* studies using murine macrophages demonstrated that lyophilized SWCNTs with glucose-PEG protectants and then held at room temperature before subsequent reconstitution maintained stable intracellular optical performance, supporting their potential for long-term storage, ease of transport, and use in biomedical applications. These findings suggest that the optimized lyophilization protocol with specific cryoprotectant combinations can significantly improve the shelf life and reproducibility of SWCNT-based sensors, paving the way for their broader application in biological and clinical settings.

## 1. Introduction

Single-walled carbon nanotubes (SWCNTs) are a class of nanomaterials characterized by their unique one-dimensional structure, described as a graphene sheet rolled seamlessly into a cylindrical shape, forming a hollow tube with an average diameter of about 1 nm [1]. This resulting structure exhibits remarkable mechanical, electrical, and optical properties [2]. SWCNTs can be uniquely characterized by their chiral indices, which are defined by a specific pair of integers denoted as (n, m) [3]. With all of their atoms effectively on the surface of the nanomaterial, SWCNTs are highly sensitive to environmental variations [4] and are ideal for a wide range of applications, particularly in the fields of biomedicine [5]. SWCNTs exhibit intrinsic fluorescence within the near-infrared (NIR) spectrum [6], specifically in the wavelength range of 900–1400 nm. This spectral range is particularly significant because it falls within the tissue transparency window [7], a region where biological tissues exhibit minimal absorption and scattering of light. As a result, NIR fluorescence from SWCNTs can effectively penetrate biological tissues, making them highly suitable for applications in biological environments [8]. Furthermore, the fluorescence emitted by SWCNTs in this range is photostable [9]. These properties are crucial for long-term imaging [10] and continuous monitoring, as they ensure consistent signal strength and reliability [11]. Consequently, SWCNTs are ideal candidates for optical biosensors, particularly *in vivo* applications [12], where stable and durable sensing capabilities are essential for monitoring biological processes within living organisms.

Due to the non-polar nature of carbon atoms, SWCNTs are naturally hydrophobic. This leads to a strong tendency for SWCNTs to aggregate, making them difficult to work with in biological and aqueous environments [8]. To address this challenge and enhance their usability, particularly in biomedical applications, SWCNTs can undergo non-covalent or covalent functionalization [13–16]. Noncovalent wrappings can include a variety of materials, ranging from surfactants [17] to biocompatible molecules such as, DNA [18–20], proteins [21], and other biopolymers [22] like PEG-lipid conjugates. By wrapping in these materials, the SWCNTs gain enhanced solubility and stability while maintaining their essential optical and electronic characteristics [8]. For biological applications, SWCNTs are commonly non-covalently functionalized with single-stranded DNA (ssDNA), where the amphiphilic ssDNA wraps the SWCNT in a helical fashion, forming a DNA-SWCNT hybrid [23,24]. The unbounded hydrophobic nitrogenous bases of ssDNA adsorb the nucleic acid to the SWCNT surface while the hydrophilic backbone of the polymer stabilizes the DNA-SWCNT hybrid by facilitating interactions with polar water molecules [25]. The wrapping and resultant stability of SWCNTs with DNA is dependent on the specific sequence of the DNA strand, in addition to pH, and other ions, small molecules, or macromolecules that may be in the immediate vicinity of the hybrid [26,27]. DNA-SWCNTs have been used in a wide variety of applications including for specific biomarker recognition [28–35].

The ability to store nanoparticles for an extended period while maintaining their stability has been an important topic in biological research for clinical applications. In the case of DNA-SWCNTs, the optimum storage and stability is crucial for their use in biomedical and sensing applications because any degradation or aggregation over time could impair their performance. Vacuum drying is the most basic form of nanoparticle storage, a process where the sample is heated to remove moisture. The nanoparticles go through one or more phase transformations during this process to end in a solid form [36], however, the method can cause significant aggregation [37]. Supercritical fluids (SCF) that exceed the critical temperature and pressure, have also been used for nanoparticle storage because they possess both liquid and gas properties such as low viscosity, high density, and ability to act as a solute for insoluble solvents [38–40]. However, this process involves thorough optimization of process parameters such as high temperature and pressure, has limited scalability, and further research is necessary to evaluate the biocompatibility of the method [41]. The most commonly employed method for long-term nanoparticle storage is lyophilization, characterized by the freezing and subsequent removal of a solvent by sublimation under vacuum [42]. Lyophilized nanoparticles maintain physical and chemical properties of the original product, have short reconstitution time, low residual moisture, long-term stability [43,44], and scalable manufacturing [45]. Muramatsu et al. demonstrated through an *in vivo* study that when lyophilized, mRNA lipid nanoparticles (mRNA-LNP) can be stored at room temperature for 12 weeks without the mRNA-LNP losing its high translatability [46]. Lyophilized products are typically rehydrated before use, allowing them to regain their original properties with minimal loss of activity or efficacy [47].

During the lyophilization process, chemical and physical stresses can be induced on the nanoparticles [48]. To stabilize the nanoparticles, two types of protectants are utilized: cryoprotectants and lyoprotectants [49]. The primary function of cryoprotectants is to protect the nanoparticles during the freezing process. Cryoprotectants cause water to melt at lower temperatures, helping to achieve regulation of the rates at which water transport, nucleation, and ice formation occur [50]. Lyoprotectants stabilize nanoparticles during both the freezing and drying process [49]. Typically, cryoprotectants can also act as lyoprotectants [49]. Various sugars are commonly used as protectants used during the lyophilization such as mannitol, sucrose, and glucose due to their ability to be vitrified during freezing [51]. Other, non-sugar protectants include dimethyl sulfoxide (DMSO), ethylene glycol, many polymers, and glycerol [52]. The type and concentration of protectant, as well as the concentration of nanoparticles impacts the degree to which the nanoparticles are stabilized [36]. In this study, we explored the use of various cryoprotectants for the long-term storage of DNA-SWCNTs. The primary objective was to enhance the stability of dispersed DNA-SWCNTs at room temperature, improve their re-dispersion after storage, and prevent unwanted aggregation, which is critical for maintaining their functionality. We systematically evaluated different cryoprotectants and optimized their ratios to identify the combination that provided the best performance in terms of intracellular stability and sensor efficacy over a four-week period. Our findings demonstrate that it is possible to store DNA-SWCNTs at room temperature for extended periods while preserving their intrinsic physical and optical properties. This advancement supports the long-term reliability of DNA-SWCNTs in various sensing applications, ensuring consistent performance over time.

## 2. Methods and materials

### 2.1. DNA-SWCNT sample preparation

To create monodispersed ssDNA wrapped SWCNTs (DNA–SWCNTs), 1 mg of HiPCO-SWCNTs was added to 2 mg of (GT)_30_ oligonucleotide (Integrated DNA Technologies) in 1 mL of 0.1 M NaCl (Sigma-Aldrich). Each sample was ultrasonicated using a 1/8″ tapered microtip for 30 min at 40 % amplitude in an ice bath (Sonics Vibracell VCX-130; Sonics and Materials). The resulting suspensions were ultra-centrifuged (Beckman Optima MAX-XP) for 30 min at 250,000 *g* and 4 °C, and the top ~80 % of the supernatant was collected.

### 2.2. Near-infrared fluorescence microscopy

A hyperspectral NIR fluorescence microscope, similar to a previously detailed system [53], was used to obtain all hyperspectral fluorescence data. A 730 nm excitation laser source was reflected onto the sample stage of an Olympus IX-73 inverted microscope equipped with a LCPlan N, 20x/0.45 IR objective by Olympus, U.S.A. The resulting fluorescence emission was passed through a volume Bragg grating and collecting with a 2D InGaAs array detector by Photon Etc. (Montreal, Canada) to generate spectral image stacks. Live cell samples were mounted on a stage top incubator by Okolab, to maintain 37 °C and 5 % CO_2_ cell culture conditions throughout the imaging procedure. All hyperspectral cubes, fluorescence images and transmitted light images were background corrected and processed in MATLAB.

### 2.3. Confocal Raman microscopy

All Raman spectroscopy data were acquired using an inverted WiTec Alpha300R confocal Raman microscope (WiTec, Germany) equipped with a Zeiss Epiplan-NEOFLUAR 100x/1.3 Oil Pol, Oil immersion, objective, a 785 nm laser (20 mW output measured at the sample), and a UHTS 300 spectrograph (300 lines/mm grating) coupled with an Andor DR32400 CCD detector (− 61C, 1650 × 200 pixels). Multiple point spectrums were scanned, and spectra were obtained using a 15s integration time and 100 accumulations per spectrum to construct hyperspectral images. Background subtraction and cosmic ray removal were performed using a polynomial function in WiTec Project 5.2 software. Hyperspectral data were extracted and processed using custom codes written with MATLAB.

### 2.4. Cell culture

RAW 264.7 TIB-71 cell line from ATCC (Manassas, VA, USA) was cultured under standard incubation conditions at 37 °C and 5 % CO2. “D-10” cell culture media containing sterile filtered high-glucose DMEM with 10 % heat inactivated FBS, 2.5 % HEPES, 1 % l-glutamine, 1 % penicillin/streptomycin, and 0.2 % amphotericin B (all from Gibco) was used for cell culture. Cells were passaged every 2–3 days and used for experiments.

### 2.5. Sample preparation for optical microscopy

For all 20x *in vitro* NIR fluorescence imaging experiments, the cells were plated, in triplicate, at an initial concentration 5.26 × 10^4^ cells/cm^2^ on 35 mm glass-bottom microwell dishes (MatTek) and allowed to culture overnight. To dose the cells with DNA-SWCNT samples, the culture media was removed and replaced with 2 mL of 1 mg-L^− 1^ GT_30_-SWCNTs diluted in D10 cell culture media and incubated for 30 min to allow cell internalization. The SWCNT-containing media was then removed, the cells were washed twice with sterile PBS (Gibco), followed by the addition of fresh media. All time points were defined with respect to this step.

### 2.6. Cryoprotectant solution preparation (sugars)

10 wt % solutions of glucose, sucrose, and mannitol were made by dissolving 10 g of each sugar in 100 mL of ultra-pure water. The solution was set to spin at 160 rpm for 1 h until all sugar was dissolved. The solutions were then passed through a 0.22 μm filter, covered with parafilm and kept in the refrigerator for experiments. Sugar solutions were only used for up to 4 days for experiments to minimize bacterial contamination.

### 2.7. Cryoprotectant solution preparation (polymers)

0.25 wt % solutions of polyethylene glycol (MW ~ 1500) and polyvinyl alcohol (MW ~ 10,000) were made in ultra-pure water. The solution was set to spin at 160 rpm for 2 h until all polymer was dissolved, covered with parafilm and kept at room temperature for experiments.

### 2.8. Lyophilization of SWCNT

SWCNT samples were added to 1 mL of each cryoprotectant solution to make up a final SWCNT concentration of 5 mg-L. The solutions were then flash frozen in a – 80 °C cryo-freezer (ThermoFisher) for 60 min. The frozen samples were then added to a lyophilizer at 0.060 Pa and – 42 °C for 24–48 h to effectively remove any moisture from the samples. Lyophilized powders were then stored at respective storage temperatures.

### 2.9. Data processing and analysis

MATLAB and OriginPro 2022b were used for data processing, background subtraction and to perform all statistical analyses. All data either met assumptions of statistical tests performed (i.e., equal variances, normality, etc.) or were transformed to meet assumptions before any statistical analysis was carried out.

## 3. Results and discussion

### 3.1. Sample properties and characterization

We synthesized DNA-SWCNTs by using standard probe-tip sonication and ultra-centrifugation, where single-stranded (GT)_30_ DNA was combined with HiPco SWCNTs, as detailed in the methods section. (GT)_30_ was chosen as the model ssDNA sequence due to its relevance in functionalizing SWCNTs in the literature [54–58], and because we expect (GT)_30_-SWCNTs to be more amenable to cryopreservation as longer oligonucleotide sequences generally exhibit better colloidal stability than their shorter counterparts [25]. This is likely due to the increased interactions between the DNA-SWCNT hybrid which is further supported by the high surface coverage of (GT)_x_ DNA oligonucleotides on the SWCNT [59]. Throughout the preparation process, meticulous attention was paid to ensure the production of a high-quality, monodispersed (GT)_30_-SWCNT dispersion, minimizing any potential for pre-existing aggregation.

Variations in SWCNT synthesis [60] as well as DNA-SWCNT functionalization can often lead to inconsistencies in sample preparation from batch to batch. Moreover, long-term room temperature storage of these biopolymer dispersions can induce DNA degradation which leads to sample aggregation and a resulting decline of NIR fluorescence properties, which can pose significant challenges for reproducibility in research [61]. To address the inherent issues of long-term storage posed by the possibility of sample aggregation over time, lyophilization was tested as a potential method to mitigate modulations in fluorescence and achieve more consistent and reproducible results. We developed an optimized lyophilization protocol specifically designed to extend the long-term shelf life of SWCNTs and considerably reduce batch-to-batch variations. Our optimized protocol involved preparing a large, homogenous batch of DNA-SWCNT dispersion, which was then lyophilized. This approach ensured that each lyophilized batch originated from the same initial dispersion, thus reducing variability.

Following sample preparation, the DNA-SWCNT dispersion was flash frozen at – 80 °C for 60 min and then lyophilized for an additional 24 h at 0.060 Pa and – 42 °C to remove moisture and produce a solid sample. The lyophilized DNA-SWCNTs were subsequently reconstituted (“L + R”) in ultra-pure water by pipette mixing and compared to the pre-lyophilization (“Pre-L”) dispersion.

**Fig. 1.**
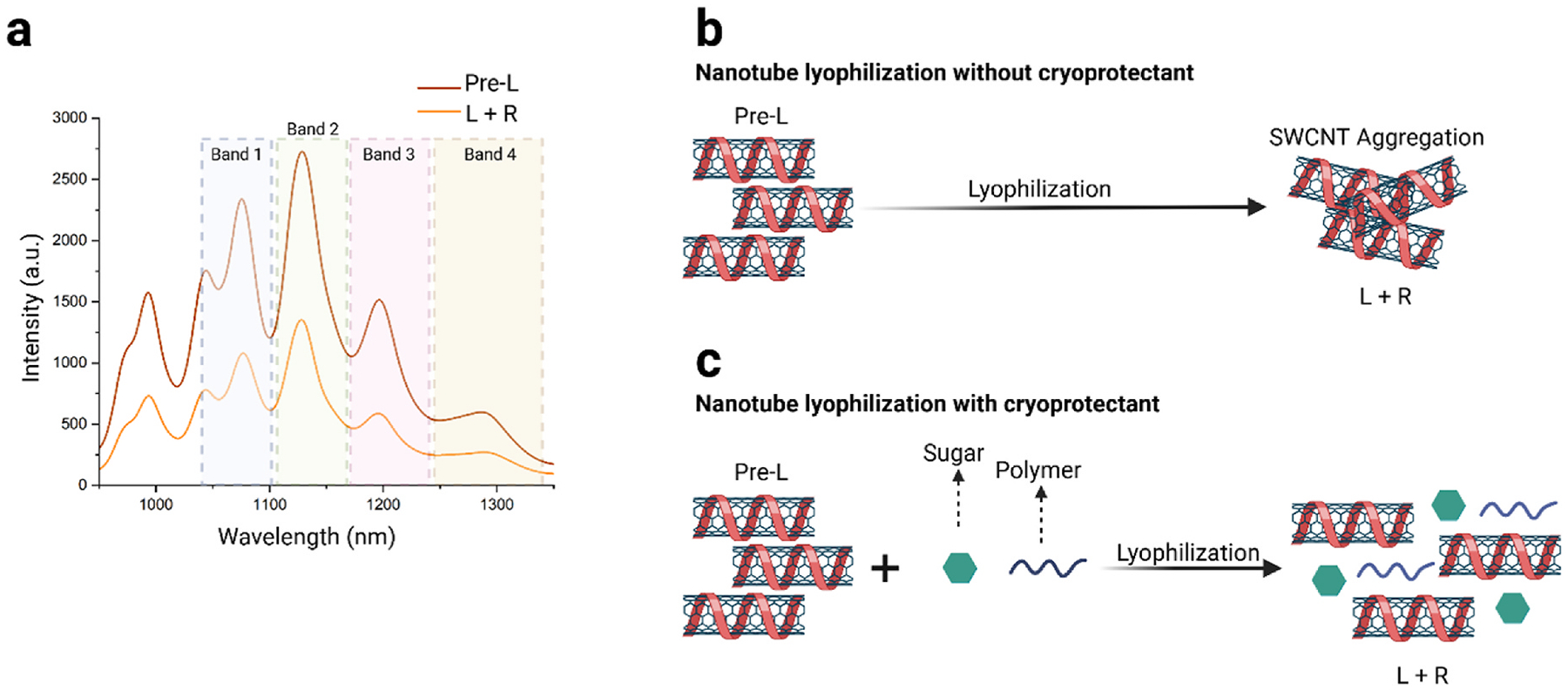
DNA-SWCNT lyophilization. (a) NIR fluorescence plot showing comparison between Pre-lyophilization (Pre-L) and lyophilization and reconstitution (L + R) of DNA-SWCNTs (no cryoprotectants added) taken under 730 nm excitation. (b) Lyophilization schematic indicating aggregation of SWCNTs (c) Proposed lyophilization schematic detailing the process of lyophilization with added cryoprotectants (CPs).

Fig. 1a illustrates the NIR fluorescence comparison between the pre-lyophilized SWCNTs (Pre-L) and the lyophilized and then reconstituted SWCNTs (L + R). The spectrum was divided into four distinct bands to aid in data analysis and comparison. The NIR fluorescence spectrum shows a substantial decrease of approximately 45 % in fluorescence intensity in the lyophilized sample (without the addition of CPs), attributable to severe aggregation. Visual comparison for both samples is shown in Movie S1 and Movie S2. The freeze-drying process has been shown to generate a variety of stresses which can in turn impact nanoparticle stability [62]. Fig. 1b provides a schematic overview of how lyophilization of SWCNTs can lead to potential aggregation and thus loss of sample properties. Fig. 1c is a proposed schematic of the process used to optimize SWCNT reconstitution after lyophilization, highlighting how the use of cryoprotectants (CPs) can potentially mitigate aggregation and improve resuspension. For this purpose, five commonly used CPs from the pharmaceutical industry were evaluated for their lyophilization effectiveness with SWCNTs (Table S1, Fig. S1) [63–66].

### 3.2. Selection of cryoprotectants for SWCNT lyophilization

Based on precedents in lyophilization formulation literature and pharmaceutical industry-standards, we selected glucose, sucrose, and mannitol, two commonly used sugars and a sugar alcohol, as well as polyethylene glycol (PEG) and polyvinyl alcohol (PVA), two polymers frequently employed in lyophilized pharmaceutical formulations, for our study [67,68,68,69]. To achieve optimal performance, the CPs were incorporated with SWCNTs and systematically investigated for many factors, such as rapid reconstitution, optical response and long-term stability. Factors considered during final SWCNT cryoprotectant additive selection are mentioned in Table S2.

**Fig. 2.**
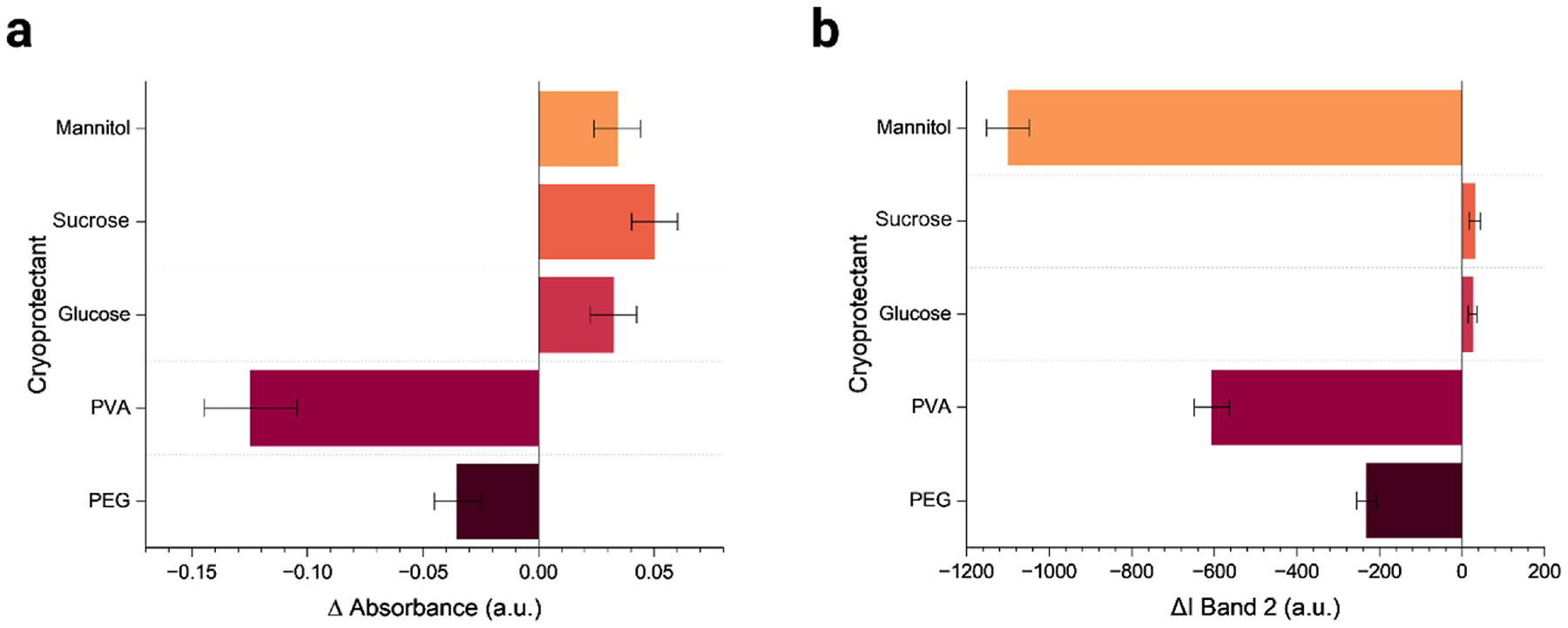
NIR characterization of lyophilized samples relative to as-dispersed DNA-SWCNTs. (a) Change in absorbance (b) Change in band 2 near infrared fluorescence peak intensity for reconstituted lyophilized samples with different CPs, each shown relative to as dispersed DNA-SWCNTs. All measurements shown are relative to as-dispersed control sample, which is set to zero, so bar values reflect the difference (Δ) between each cryoprotectant condition and the as-dispersed sample. Error bars represent mean±st.dev. from n = 3 technical replicates.

A freshly prepared DNA-SWCNT sample was added to 1 mL of each selected CPs to achieve a final SWCNT concentration of 5 mg-L^− 1^ and the absorbance of each sample was measured (Fig. S2). No appreciable changes in the absorbance spectrum with respect to the as-dispersed SWCNT reference were observed for DNA-SWCNT samples upon addition of CPs, lyophilization and reconstitution, with the exception of PVA (Fig. 2a, Fig. S2e). A detailed table of absorbance change observed at 990 nm in SWCNT (L + R) samples with respect to the as-dispersed SWCNT sample is shown in Table S3. Absorbance data suggest glucose and sucrose show positive trends in absorbance recovery after reconstitution, indicating effective cryopreservation upon reconstitution (Fig. S3). PEG also performs well upon reconstitution and absorbance data of the SWCNT (L + R) samples containing mannitol indicate similar reconstitution performance, whereas PVA shows a significant decrease in intensity with a degradation of nearly 57 % in absorbance and sample quality upon reconstitution. The trends observed for changes in absorbance after reconstitution of lyophilized samples are similar to changes in NIR fluorescence of Band 2 for (L + R) samples, with respect to the as-dispersed SWCNT sample, except for mannitol (Fig. 2b). We attribute mannitol’s response to the presence of small bundles of aggregates (Fig. S4) that were not detectable by absorbance but still result in a decreased fluorescence intensity, which is a significantly more sensitive optical reporter as reported in literature [70]. Similar data for the other NIR fluorescence band intensities and the respective wavelength ranges considered during analysis of data (Fig. S5) can be found in Table S4.

A considerable decrease in band 2 intensity was noted with the PVA and mannitol samples, exhibiting a significant 27 % and 50 % reduction in fluorescence respectively, compared to the as-dispersed sample. In contrast, the sample with PEG resulted in a 10 % decrease in fluorescence intensity. Glucose and sucrose samples performed well in NIR fluorescence quality checks with both giving the most consistent performance with only a 1 % increase in intensity. In summary, the data show considerable promise in performance and quality of samples for all CPs used except for PVA and mannitol. The poor performance of PVA as a CP has also been reported for gold nanoparticles [71] and, in our DNA-SWCNT system, can likely be attributed to strong interactions between PVA and the adsorbed (GT)_30_ ssDNA molecule which is preferentially complexing with the PVA polymer [72], thus leading to SWCNT aggregation (Fig. S6).

To reach an informed decision regarding the quality and performance of all DNA-SWCNT (L + R) samples, lyophilized powders of each sample were analyzed via Raman microscopy (Fig. S7) and Fourier transform infrared (FTIR) spectroscopy (Figs. S8 and S9) to elucidate qualitative structural information. Traditionally, Raman microscopy of SWCNTs focuses on analyzing the G-band (1585 cm^− 1^), which scales linearly with the concentration of SWCNTs, and the D-band (1310–1350 cm^− 1^), also known as the disorder or defect band, which quantifies defects on the nanotube surface [73]. In this study, Raman spectrum sharpness obtained from Raman microscopy was utilized to qualitatively identify the crystalline structure [74] of the DNA-SWCNT samples with cryoprotectants. The Raman spectrum for glucose- and sucrose-containing samples showed broad bands, indicative of their amorphous nature. In contrast, the spectrum for PEG containing sample exhibited well-defined sharp peaks, reflecting strong crystalline structure [75]. The PVA sample showed indications for a higher D/G ratio. This could potentially be due to the lyophilization process on a colloidally unstable suspension trapping SWCNTs in a bent conformation [76]. Previous literature shows that the freeze-drying process can directly damage nanoparticles if not processed with the correct choice of cryoprotectants, which can cause loss of stability during flash freezing that can lead to particle aggregation [77–79]. Similar conclusions can be observed in the FTIR results (Figs. S8 and S9), where the PEG sample presents well-defined peaks at its characteristic fingerprint bands in contrast to the sugar samples that have broad peaks in comparison especially at the O–H band indicative of a more amorphous structure [80–82].

All CPs were further tested for in-vitro compatibility as shown in Figs. S10 and S11. It was observed that PVA was not a good choice as CP for our system due to aggregation.

Based on all characterization results, glucose and PEG were selected as the most suitable CP candidates for the lyophilization of SWCNTs, a choice which is consistent with previous literature on the use of cryoprotectants when lyophilizing nanoparticles [83,84]. In contrast, PVA displayed a high degree of aggregation when lyophilized with SWCNTs, negatively affecting reconstitution efficiency, as well as the absorbance and NIR fluorescence responses. Sucrose and mannitol were not pursued further due to complications regarding polymorphism which can induce batch to batch variability leading to stability issues, and other potential complications in downstream biomedical applications [85,86].

### 3.3. Evaluating cryoprotectant ratios for optimum performance

Additional investigations into various weight ratios of glucose:PEG were conducted to identify the optimal combination for achieving the best long-term results as previous work has shown that combinations of multiple cryoprotectants have shown promise in exhibiting superior cryoprotection [87].

**Fig. 3.**
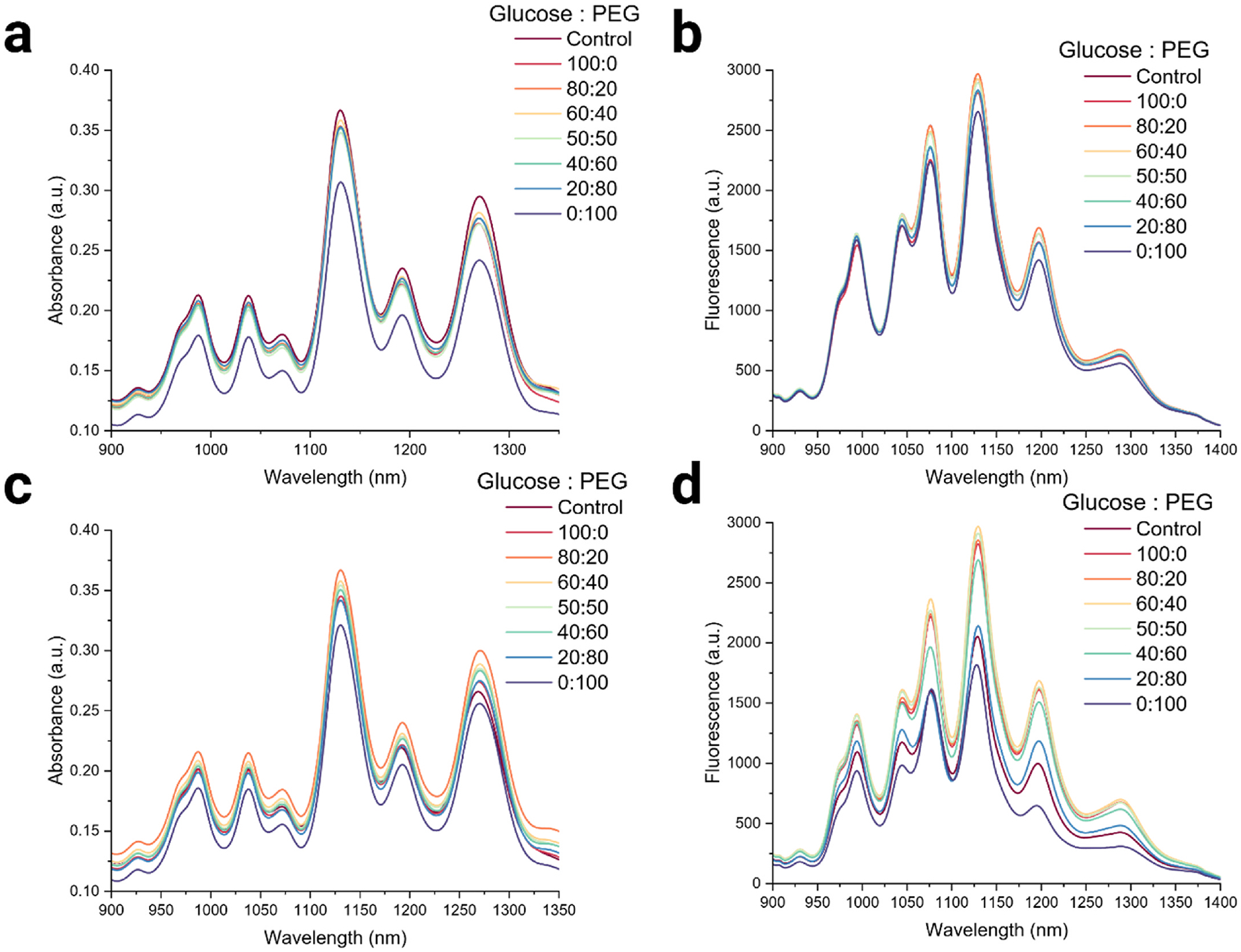
Evaluating differential weight ratios of glucose:PEG. (a) Absorbance spectroscopy plot comparing spectrum of DNA-SWCNT samples with different weight ratios of glucose:PEG pre-lyophilization and control SWCNTs with no CPs. (b) NIR fluorescence spectra of DNA-SWCNT samples with different ratios of glucose:PEG pre-lyophilization and control SWCNTs with no CPs (c) Absorbance spectroscopy spectra comparing DNA-SWCNT samples with different ratios of glucose:PEG post-lyophilization and reconstitution & control SWCNTs with no CPs. (d) NIR fluorescence plot of DNA-SWCNT samples with different ratios of glucose:PEG post-lyophilization & reconstitution and control SWCNTs with no CPs.

Fig. 3a and b represent the pre-lyophilization absorbance spectroscopy and NIR fluorescence results for the different CP ratios of glucose and PEG tested. In the pre-lyophilization stage, no significant differences were observed across the CP weight ratios, except for the combination containing 0 % glucose and 100 % PEG. The different weight ratios were then lyophilized and reconstituted, and the absorbance spectra measured (Fig. 3c). All samples containing CPs performed better than the reconstituted control (no CP) sample, except for the sample with 100 % PEG. The NIR fluorescence spectra for all lyophilized and reconstituted samples were measured (Fig. 3d), and a trend emerged that higher concentrations of PEG resulted in a decrease in the average fluorescence intensity. We hypothesize that at higher concentrations of PEG, the polymer acts as a macromolecular crowding agent that can interact with the exposed hydrophilic ssDNA backbone, reducing the free-energy advantage of DNA adsorption on SWCNTs; this leads to partial ssDNA desorption and loss of DNA-SWCNT hybrid stabilization, allowing depletion attractions to drive SWCNT bundling and photo-luminescence quenching [88–92]. The weight ratios of 80:20, 60:40, and 50:50 showed the best performance and were therefore selected for further long-term and *in vitro* cell studies. We further hypothesize that glucose and PEG act synergistically because each contributes a distinct and complementary stabilization mechanism during freezing and drying [93,94]. Glucose primarily serves as a hydrogen-bonding vitrifying agent that replaces structural water around the DNA-SWCNT hybrid and suppresses ice-induced mechanical stress [95–97]. Low levels of PEG likely contribute by increasing viscosity and inhibiting ice crystallization without generating the macromolecular crowding forces that at higher PEG fractions disfavor ssDNA adsorption and promote depletion-driven SWCNT bundling. Thus, for formulations with both glucose and PEG, the cryoprotectants act synergistically to maintain the DNA-SWCNT supramolecular complex in a vitrified, kinetically trapped state while avoiding the wrapping displacement and aggregation observed with PEG-rich compositions. Although the sample with 100 % glucose also exhibited satisfactory NIR fluorescence spectra, it was not chosen for any further investigations due to characterization results indicating the amorphous nature of the lyophilized DNA-SWCNT sample with glucose as cryoprotectant. Literature shows that for long term storage a more crystalline nanoparticle product is preferred due to improved thermodynamic stability and structural integrity. Although many drugs benefit from an amorphous structure that leads to higher dissolution rates and apparent solubility thus increasing bioavailability, but such advantages are usually tempered by their lack of long-term stability and shelf life. Crystalline structures are therefore preferred as they offer greater stability [64,98].

### 3.4. In-vitro optical performance

**Fig. 4.**
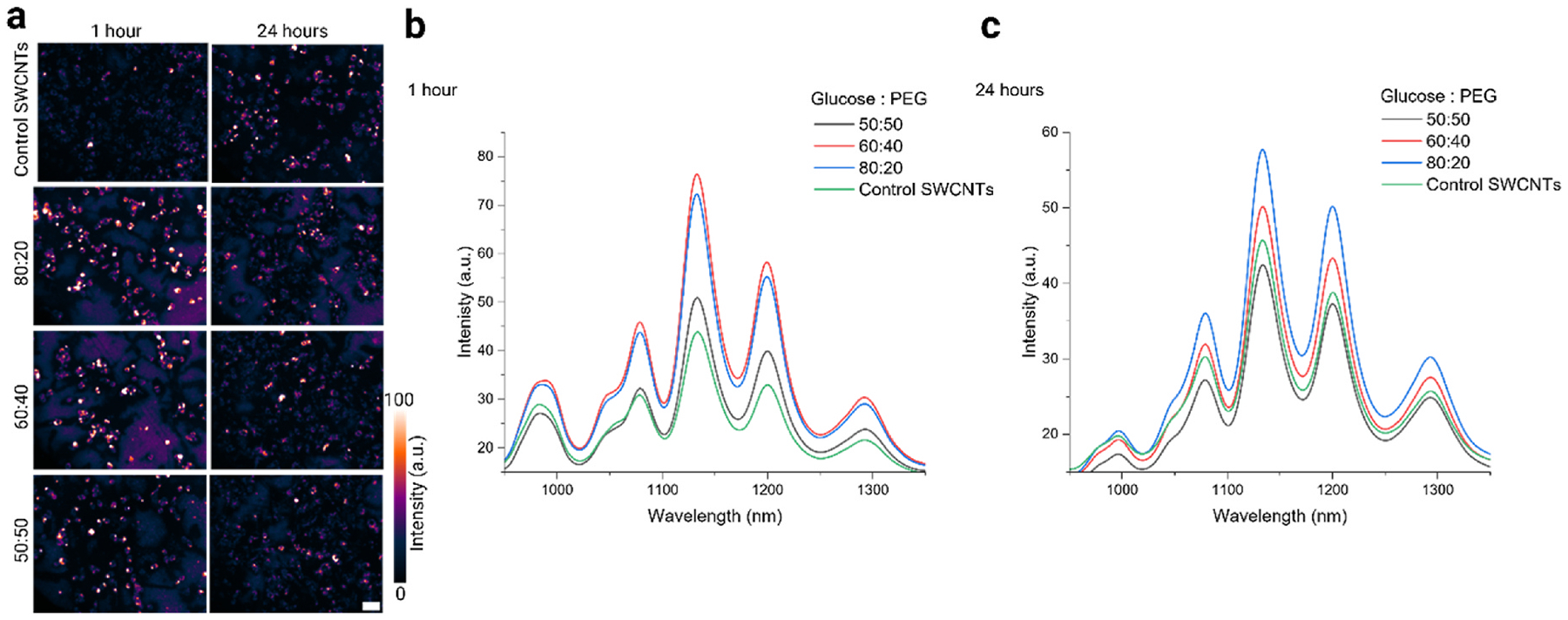
*In vitro* cell investigations of selected ratio L + R samples. (a) NIR broadband fluorescence (900–1400 nm) images of all selected ratio samples and the as dispersed control sample incubated with murine macrophages. Images have been globally contrasted to the brightest image. Scale bar = 15 μm (b) NIR fluorescence spectral response of cells incubated with selected SWCNTs (L + R) CP ratios and as-dispersed control sample at 1 h and (c) 24 h.

We assessed the internalization and *in vitro* optical performance of the selected ratio L + R samples in live cultured cells. Macrophages (murine RAW 264.7), which are crucial components of the immune system and serve as the body’s first line of defense [99], were chosen and could be compared to our own and other groups’ previous studies [28,61,68–100]. The cultured macrophages were incubated with 1 mg-L^− 1^ of each selected ratio of glucose-PEG (L + R) and a GT_30_ SWCNT control sample (no CPs) for 30 min. Following this pulse incubation, the cells were thoroughly washed with 1x PBS (phosphate-buffered saline) and placed in fresh media for an additional 30 min before imaging at the 1-h time point. Here, the as-dispersed DNA-SWCNTs were used as standard control for all intracellular experiments. Fig. 4a shows the intracellular broadband fluorescent images for all samples and distinct changes in both 1 h and 24 h can be observed. Fig. 4b and c shows NIR broadband fluorescence spectral response of the images in 4a. As expected, all glucose-PEG ratio samples performed better than the as-dispersed DNA-SWCNT control. The 80:20 and 60:40 glucose: PEG sample showed a larger increase in intensity relative to control, while the behavior of 50:50 sample was similar in nature to the as-dispersed SWCNTs. The increased fluorescence performance of these glucose and PEG ratios is attributed to increased cellular uptake.

We speculate that including glucose as a cryoprotectant molecules increases nanoparticle uptake in phagocytic cells such as macrophages because phagocytosis is a glucose-mediated cellular process [101,102]; this hypothesis is supported by data demonstrating that there was an increased fluorescence response of non-lyophilized SWCNTs inside macrophages when incubated with glucose-enriched media (Fig. S12). Interestingly, there was also an increase in intracellular fluorescence when macrophages were incubated with non-lyophilized SWCNTs in PEG-doped media, a result which could merit future investigation as PEG is canonically a bio-inert polymer [103] (Fig. S12).

### 3.5. Long term stability investigations

**Fig. 5.**
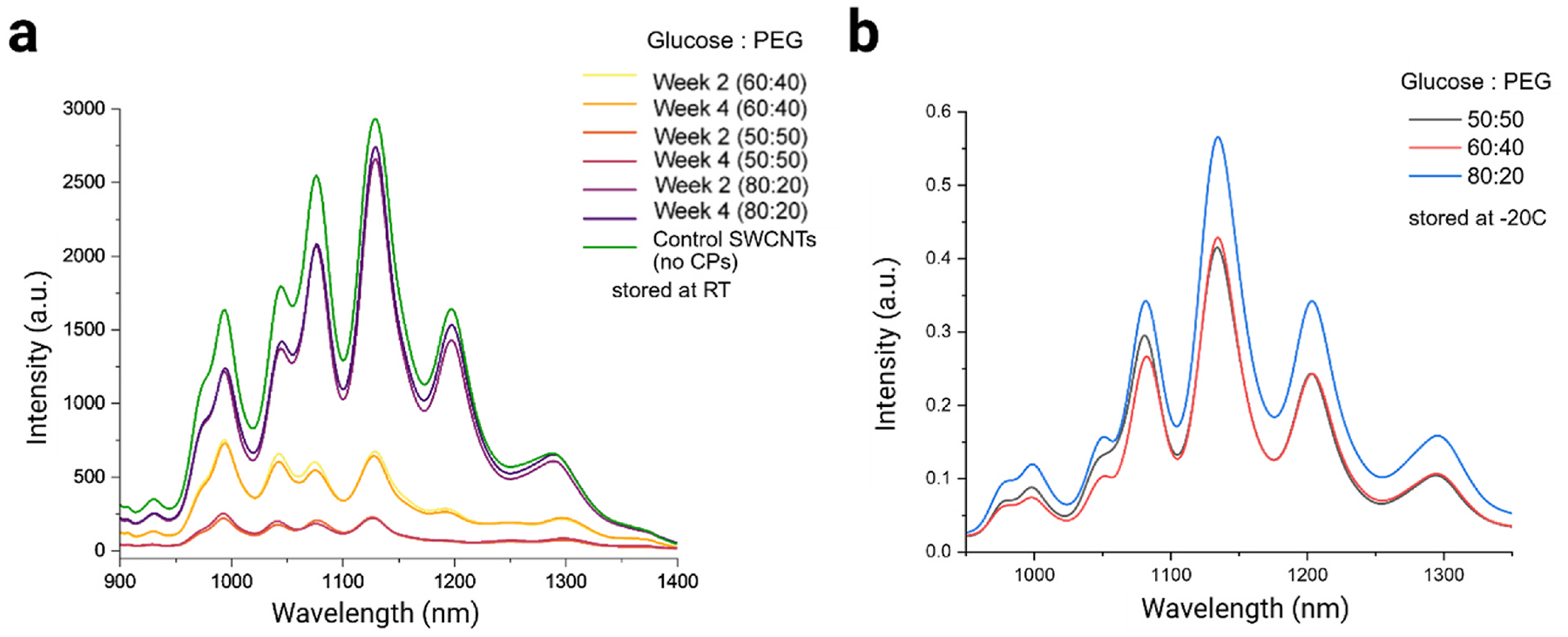
Long-term stability assessment of lyophilized DNA-SWCNTs stored at room temperature. (a) NIR fluorescence stability of lyophilized and reconstituted samples compared to control (as-dispersed) SWCNTs stored at room temperature for 2 and 4 weeks. (b) NIR fluorescence stability (normalized) of lyophilized and reconstituted (L + R) samples compared to control (as dispersed) SWCNTs stored at – 20°C for more than 12 months.

For long-term storage studies, the selected samples were analyzed for their NIR fluorescence response after storage for 2 or 4 weeks at three different temperatures: room temperature (RT, approximately 22 °C), – 20 °C, and – 80 °C (Fig. S13). For the samples stored at room temperature, both the 60:40 and 50:50 glucose-to-PEG ratios showed a notable decrease in NIR fluorescence, with more than 50 % reduction and optical performance degradation in integrated NIR fluorescence intensity signal compared to the as-dispersed DNA-SWCNT (Fig. 5a). In contrast, the 80:20 sample remained highly stable across all storage temperatures, including room temperature. We also observed that the NIR fluorescence at room temperature was quite stable between week 2 and week 4 for all the samples, meaning any degradation to quality of samples occurred within the first week. All samples stored at – 20 °C and – 80 °C exhibited similar responses, with no major differences observed (Fig. S13) which is to be expected of lyophilized components under cold-chain storage. Interestingly, the 80:20 glucose-to-PEG DNA-SWCNT sample remained relatively stable for more than 12 months at room temperature and – 20°C as compared to the other lyophilized samples, showing limited signs of degradation (Fig. 5b and Fig. S14). and aggregation (Movie S3–S5).

Finally, to assess the ability of lyophilized and reconstituted DNA-SWCNTs to retain their sensing abilities, their response to hydrogen peroxide was examined and compared to as-dispersed DNA-SWCNT sample (Fig. S15). Notably, the DNA-SWCNT L + R sample with 80:20 glucose:PEG ratio still responded to the addition of 2 mM hydrogen peroxide in a similar fashion as that of the as-dispersed DNA-SWCNT sample. To establish generalizability of our lyophilization protocol and reaffirm that it was translatable to different chirality-enriched samples, SG65i and SG76i were investigated (Fig. S16). The findings demonstrate that our optimized lyophilization procedure is broadly applicable to other chirality-enriched species as well, highlighting its versatility for sample preparation, storage and stability in carbon nanomaterial research workflows.

## 4. Conclusion

The objective of this study was to systematically evaluate different CPs as potential additives to DNA-SWCNT samples to achieve more consistent, reproducible, and optimized outcomes with long-term stability. Five CPs were investigated, along with various ratios of the most effective candidates. Glucose and PEG were selected as the primary CPs due to their optimized optical performance, stability, and compatibility in *in vitro* cell studies. Further research showed that the 80:20 wt ratio of glucose:PEG offered the most optimized response for both intracellular optical performance and applications targeting the sensing of specific biomolecules. The 80:20 glucose:PEG ratio proved to be the most stable, performing best overall, and could be stored at room temperature and – 20°C without significant degrading the DNA wrapping or stability for more than 12 months. Lyophilizing SWCNTs specifically with 80:20 glucose:PEG ratio not only enhances practical applicability for long term storage but potentially revolutionizes storage and long-term handling of biopolymer suspended SWCNTs specifically for biomedical applications like biosensing and diagnostic tool development. This could in turn lead to more reliable and efficient technologies, significantly impacting healthcare outcomes by enabling more precise detection and monitoring.

## Supplementary Material

Supporting Information text

SI Movie 1

SI Movie 2

SI Movie 3

SI Movie 4

SI Movie 5

## Data Availability

Data will be made available on request.

## Appendix A. Supplementary data

Supplementary data to this article can be found online at https://doi.org/10.1016/j.carbon.2025.121159.

## References

[1] M. N, S.K.V. P, S.M. S, R. G, V.S. K, T.K S Carbon nanotubes and their properties-the review, Mater. Today Proc 47 (2021) 4682–4685, 10.1016/j.matpr.2021.05.543.

[2] Thostenson ET, Ren Z, Chou T-W, Advances in the science and technology of carbon nanotubes and their composites: a review, Compos. Sci. Technol 61 (13) (2001) 1899–1912, 10.1016/S0266-3538(01)00094-X.

[3] Zheng M, Sorting carbon nanotubes, Top. Curr. Chem 375 (1) (2017) 13, 10.1007/s41061-016-0098-z. 28083771

[4] Kruss S, Landry MP, Vander Ende E, Lima BMA, Reuel NF, Zhang J, Nelson J, Mu B, Hilmer A, Strano M, Neurotransmitter detection using Corona phase molecular recognition on fluorescent single-walled carbon nanotube sensors, J. Am. Chem. Soc 136 (2) (2014) 713–724, 10.1021/ja410433b. 24354436

[5] Farrera C, Torres Andón F, Feliu N, Carbon nanotubes as optical sensors in biomedicine, ACS Nano 11 (11) (2017) 10637–10643, 10.1021/acsnano.7b06701. 29087693

[6] O’Connell MJ, Bachilo SM, Huffman CB, Moore VC, Strano MS, Haroz EH, Rialon KL, Boul PJ, Noon WH, Kittrell C, Ma J, Hauge RH, Weisman RB, Smalley RE, Band gap fluorescence from individual single-walled carbon nanotubes, Science 297 (5581) (2002) 593–596, 10.1126/science.1072631. 12142535

[7] Zhao J, Zhong D, Zhou S, NIR-I-to-NIR-II fluorescent nanomaterials for biomedical imaging and cancer therapy, J. Mater. Chem. B 6 (3) (2018) 349–365, 10.1039/C7TB02573D. 32254515

[8] Ackermann J, Metternich JT, Herbertz S, Kruss S, Biosensing with fluorescent carbon nanotubes, Angew. Chem. Int. Ed 61 (18) (2022), 10.1002/anie.202112372. PMC9313876 34978752

[9] Boghossian AA, Zhang J, Barone PW, Reuel NF, Kim J, Heller DA, Ahn J, Hilmer AJ, Rwei A, Arkalgud JR, Zhang CT, Strano MS, Near-infrared fluorescent sensors based on single-walled carbon nanotubes for life sciences applications, ChemSusChem 4 (7) (2011) 848–863, 10.1002/cssc.201100070. 21751417

[10] Heller DA, Baik S, Eurell TE, Strano MS, Single-walled carbon nanotube spectroscopy in live cells: towards long-term labels and optical sensors, Adv. Mater 17 (23) (2005) 2793–2799, 10.1002/adma.200500477.

[11] Coleman JN, Liquid-phase exfoliation of nanotubes and graphene, Adv. Funct. Mater 19 (23) (2009) 3680–3695, 10.1002/adfm.200901640.

[12] Giraldo JP, Landry MP, Kwak S, Jain RM, Wong MH, Iverson NM, Ben-Naim M, Strano MS, A ratiometric sensor using single chirality near-infrared fluorescent carbon nanotubes: application to in vivo monitoring, Small 11 (32) (2015) 3973–3984, 10.1002/smll.201403276. 25981520

[13] Oliveira SF, Bisker G, Bakh NA, Gibbs SL, Landry MP, Strano MS, Protein functionalized carbon nanomaterials for biomedical applications, Carbon N Y 95 (2015) 767–779, 10.1016/j.carbon.2015.08.076.

[14] Karousis N, Tagmatarchis N, Tasis D, Current progress on the chemical modification of carbon nanotubes, Chem Rev 110 (9) (2010) 5366–5397, 10.1021/cr100018g. 20545303

[15] Kallmyer NE, Huynh T, Graves JC, Musielewicz J, Tamiev D, Reuel NF, Influence of sonication conditions and wrapping type on yield and fluorescent quality of noncovalently functionalized single-walled carbon nanotubes, Carbon N Y 139 (2018) 609–613, 10.1016/j.carbon.2018.07.028.

[16] Narimatsu K, Nishioka J, Murakami H, Nakashima N, Design, synthesis, and characterization of carbon nanotube solubilizers carrying a reactive group, Chem. Lett 35 (8) (2006) 892–893, 10.1246/cl.2006.892.

[17] Moore VC, Strano MS, Haroz EH, Hauge RH, Smalley RE, Schmidt J, Talmon Y, Individually suspended single-walled carbon nanotubes in various surfactants, Nano Lett. 3 (10) (2003) 1379–1382, 10.1021/nl034524j.

[18] Card M, Alejandro R, Roxbury D, Decoupling individual optical nanosensor responses using a spin-coated hydrogel platform, ACS Appl. Mater. Interfaces 15 (1) (2023) 1772–1783, 10.1021/acsami.2c16596. 36548478

[19] Safaee MM, Gravely M, Rocchio C, Simmeth M, Roxbury D, DNA sequence mediates apparent length distribution in single-walled carbon nanotubes, ACS Appl. Mater. Interfaces 11 (2) (2019) 2225–2233, 10.1021/acsami.8b16478. 30575397

[20] Card M, Gravely M, Madani SZM, Roxbury D, A spin-coated hydrogel platform enables accurate investigation of immobilized individual single-walled carbon nanotubes, ACS Appl. Mater. Interfaces 13 (27) (2021) 31986–31995, 10.1021/acsami.1c06562. 34197074

[21] Antonucci A, Kupis-Rozmysłowicz J, Boghossian AA, Noncovalent protein and peptide functionalization of single-walled carbon nanotubes for biodelivery and optical sensing applications, ACS Appl. Mater. Interfaces 9 (13) (2017) 11321–11331, 10.1021/acsami.7b00810. 28299937

[22] Gillen AJ, Boghossian AA, Non-covalent methods of engineering optical sensors based on single-walled carbon nanotubes, Front. Chem 7 (2019), 10.3389/fchem.2019.00612. PMC6763700 31616652

[23] Veetil JV, Ye K, Tailored carbon nanotubes for tissue engineering applications, Biotechnol. Prog 25 (3) (2009) 709–721, 10.1002/btpr.165. 19496152 PMC2700190

[24] Rajendra J, Baxendale M, Dit Rap LG, Rodger A, Flow linear dichroism to probe binding of aromatic molecules and DNA to single-walled carbon nanotubes, J. Am. Chem. Soc 126 (36) (2004) 11182–11188, 10.1021/ja048720j. 15355099

[25] Gravely M, Safaee MM, Roxbury D, Biomolecular functionalization of a nanomaterial to control stability and retention within live cells, Nano Lett. 19 (9) (2019) 6203–6212, 10.1021/acs.nanolett.9b02267. 31424226 PMC7199458

[26] Landry MP, Vuković L, Kruss S, Bisker G, Landry AM, Islam S, Jain R, Schulten K, Strano MS, Comparative dynamics and sequence dependence of DNA and RNA binding to single walled carbon nanotubes, J. Phys. Chem. C 119 (18) (2015) 10048–10058, 10.1021/jp511448e. PMC4440682 26005509

[27] Xhyliu F, Ao G, Chirality-pure carbon nanotubes show distinct complexation with recognition DNA sequences, Carbon N Y 167 (2020) 601–608, 10.1016/j.carbon.2020.06.040.

[28] Madani SZM, Safaee MM, Gravely M, Silva C, Kennedy S, Bothun GD, Roxbury D, Carbon nanotube–liposome complexes in hydrogels for controlled drug delivery via near-infrared laser stimulation, ACS Appl. Nano Mater 4 (1) (2021) 331–342, 10.1021/acsanm.0c02700.

[29] Safaee MM, Gravely M, Roxbury D, A wearable optical microfibrous biomaterial with encapsulated nanosensors enables wireless monitoring of oxidative stress, Adv. Funct. Mater 31 (13) (2021), 10.1002/adfm.202006254.

[30] Gravely M, Roxbury D, Multispectral fingerprinting resolves dynamics of nanomaterial trafficking in primary endothelial cells, ACS Nano 15 (7) (2021) 12388–12404, 10.1021/acsnano.1c04500. 34180232

[31] Yaari Z, Yang Y, Apfelbaum E, Cupo C, Settle AH, Cullen Q, Cai W, Roche KL, Levine DA, Fleisher M, Ramanathan L, Zheng M, Jagota A, Heller DA, A perception-based nanosensor platform to detect cancer biomarkers, Sci. Adv 7 (47) (2021), 10.1126/sciadv.abj0852. PMC8604403 34797711

[32] Nadeem A, Lyons S, Kindopp A, Jamieson A, Roxbury D, Machine learning-assisted near-infrared spectral fingerprinting for macrophage phenotyping, ACS Nano 18 (34) (2024) 22874–22887, 10.1021/acsnano.4c03387. 39148286 PMC12020776

[33] Bhattacharya S, Gong X, Wang E, Dutta SK, Caplette JR, Son M, Nguyen FT, Strano MS, Mukhopadhyay D, DNA–SWCNT biosensors allow real-time monitoring of therapeutic responses in pancreatic ductal adenocarcinoma, Cancer Res. 79 (17) (2019) 4515–4523, 10.1158/0008-5472.CAN-18-3337. 31292162 PMC6726513

[34] Yoon M, Lee Y, Lee S, Cho Y, Koh D, Shin S, Tian C, Song Y, Kang J, Cho S-Y, A NIR fluorescent single walled carbon nanotube sensor for broad-spectrum diagnostics, Sensors & Diagnostics 3 (2) (2024) 203–217, 10.1039/D3SD00257H.

[35] Jin C, Qiu L, Li J, Fu T, Zhang X, Tan W, Cancer biomarker discovery using DNA aptamers, Analyst 141 (2) (2016) 461–466, 10.1039/C5AN01918D. 26567694 PMC4701631

[36] Degobert G, Aydin D, Lyophilization of nanocapsules: instability sources, formulation and process parameters, Pharmaceutics 13 (8) (2021) 1112, 10.3390/pharmaceutics13081112. 34452072 PMC8400524

[37] Zhao P, Hou X, Yan J, Du S, Xue Y, Li W, Xiang G, Dong Y, Long-term storage of lipid-like nanoparticles for MRNA delivery, Bioact. Mater 5 (2) (2020) 358–363, 10.1016/j.bioactmat.2020.03.001. 32206737 PMC7078456

[38] Su W, Zhang H, Xing Y, Li X, Wang J, Cai C, A bibliometric analysis and review of supercritical fluids for the synthesis of nanomaterials, Nanomaterials 11 (2) (2021) 336, 10.3390/nano11020336. 33525541 PMC7910895

[39] Byrappa K, Ohara S, Adschiri T, Nanoparticles synthesis using supercritical fluid technology – towards biomedical applications, Adv. Drug Deliv. Rev 60 (3) (2008) 299–327, 10.1016/j.addr.2007.09.001. 18192071

[40] Campardelli R, Trucillo P, Reverchon E, A supercritical fluid-based process for the production of fluorescein-loaded liposomes, Ind. Eng. Chem. Res 55 (18) (2016) 5359–5365, 10.1021/acs.iecr.5b04885.

[41] O’Sullivan A, Ryan KM, Padrela L, Production of biopharmaceutical dried-powders using supercritical CO2 technology, J. Supercrit. Fluids 187 (2022) 105645, 10.1016/j.supflu.2022.105645.

[42] Chen C, Han D, Cai C, Tang X, An overview of liposome lyophilization and its future potential, J. Contr. Release 142 (3) (2010) 299–311, 10.1016/j.jconrel.2009.10.024. 19874861

[43] Ball R, Bajaj P, Whitehead K, Achieving long-term stability of lipid nanoparticles: examining the effect of PH, temperature, and lyophilization, Int J Nanomedicine 12 (2016) 305–315, 10.2147/IJN.S123062. 28115848 PMC5221800

[44] Kasper JC, Winter G, Friess W, Recent advances and further challenges in lyophilization, Eur. J. Pharm. Biopharm 85 (2) (2013) 162–169, 10.1016/j.ejpb.2013.05.019. 23751601

[45] Colby AH, Liu R, Doyle RP, Merting A, Zhang H, Savage N, Chu N-Q, Hollister BA, McCulloch W, Burdette JE, Pearce CJ, Liu K, Oberlies NH, Colson YL, Grinstaff MW, Pilot-scale production of expansile nanoparticles: practical methods for clinical Scale-Up, J. Contr. Release 337 (2021) 144–154, 10.1016/j.jconrel.2021.07.012. PMC8489532 34280414

[46] Muramatsu H, Lam K, Bajusz C, Laczkó D, Karikó K, Schreiner P, Martin A, Lutwyche P, Heyes J, Pardi N, Lyophilization provides long-term stability for a lipid nanoparticle-formulated, nucleoside-modified MRNA vaccine, Mol. Ther 30 (5) (2022) 1941–1951, 10.1016/j.ymthe.2022.02.001. 35131437 PMC8815268

[47] Karunnanithy V, Abdul Rahman NHB, Abdullah NAH, Fauzi MB, Lokanathan Y, Min Hwei AN, Maarof M, Effectiveness of lyoprotectants in protein stabilization during lyophilization, Pharmaceutics 16 (10) (2024) 1346, 10.3390/pharmaceutics16101346. 39458674 PMC11510631

[48] Abdelwahed W, Degobert G, Fessi H, Investigation of nanocapsules stabilization by amorphous excipients during freeze-drying and storage, Eur. J. Pharm. Biopharm 63 (2) (2006) 87–94, 10.1016/j.ejpb.2006.01.015. 16621490

[49] Fonte P, Reis S, Sarmento B, Facts and evidences on the lyophilization of polymeric nanoparticles for drug delivery, J. Contr. Release 225 (2016) 75–86, 10.1016/j.jconrel.2016.01.034. 26805517

[50] Yang J, Cai N, Zhai H, Zhang J, Zhu Y, Zhang L, Natural zwitterionic betaine enables cells to survive ultrarapid cryopreservation, Sci. Rep 6 (1) (2016) 37458, 10.1038/srep37458. 27874036 PMC5118695

[51] Wang W, Lyophilization and development of solid protein pharmaceuticals, Int J Pharm 203 (1–2) (2000) 1–60, 10.1016/S0378-5173(00)00423-3. 10967427

[52] Boafo GF, Magar KT, Ekpo MD, Qian W, Tan S, Chen C, The role of cryoprotective agents in liposome stabilization and preservation, Int. J. Mol. Sci 23 (20) (2022) 12487, 10.3390/ijms232012487. 36293340 PMC9603853

[53] Roxbury D, Jena PV, Shamay Y, Horoszko CP, Heller DA, Cell membrane proteins modulate the carbon nanotube optical bandgap via surface charge accumulation, ACS Nano 10 (1) (2016) 499–506, 10.1021/acsnano.5b05438. 26654246 PMC4975035

[54] Zheng Y, Alizadehmojarad AA, Bachilo SM, Weisman RB, Guanine-specific chemical reaction reveals SsDNA interactions on carbon nanotube surfaces, J. Phys. Chem. Lett 13 (9) (2022) 2231–2236, 10.1021/acs.jpclett.2c00030. 35238575

[55] Zheng M, Jagota A, Semke ED, Diner BA, Mclean RS, Lustig SR, Richardson RE, Tassi NG, DNA-assisted dispersion and separation of carbon nanotubes, Nat. Mater 2 (5) (2003) 338–342, 10.1038/nmat877. 12692536

[56] Zheng M, Jagota A, Strano MS, Santos AP, Barone P, Chou SG, Diner BA, Dresselhaus MS, Mclean RS, Onoa GB, Samsonidze GG, Semke ED, Usrey M, Walls DJ, Structure-based carbon nanotube sorting by sequence-dependent DNA assembly, Science 302 (5650) (2003) 1545–1548, 10.1126/science.1091911. 14645843

[57] Nißler R, Mann FA, Chaturvedi P, Horlebein J, Meyer D, Vuković L, Kruss S, Quantification of the number of adsorbed DNA molecules on single-walled carbon nanotubes, J. Phys. Chem. C 123 (8) (2019) 4837–4847, 10.1021/acs.jpcc.8b11058.

[58] Loewenthal D, Kamber D, Bisker G, Monitoring the activity and inhibition of cholinesterase enzymes using single-walled carbon nanotube fluorescent sensors, Anal. Chem 94 (41) (2022) 14223–14231, 10.1021/acs.analchem.2c02471. 36206351 PMC9583068

[59] Alizadehmojarad AA, Bachilo SM, Weisman RB, Sequence-dependent surface coverage of SsDNA coatings on single-wall carbon nanotubes, J. Phys. Chem. A 128 (28) (2024) 5578–5585, 10.1021/acs.jpca.4c02809. 38981061

[60] Mansfield E, Kar A, Hooker SA, Applications of TGA in quality control of SWCNTs, Anal. Bioanal. Chem 396 (3) (2010) 1071–1077, 10.1007/s00216-009-3319-2. 20016881

[61] Grass RN, Heckel R, Puddu M, Paunescu D, Stark WJ, Robust chemical preservation of digital information on DNA in silica with error-correcting codes, Angew. Chem. Int. Ed 54 (8) (2015) 2552–2555, 10.1002/anie.201411378. 25650567

[62] Trenkenschuh E, Friess W, Freeze-drying of nanoparticles: how to overcome colloidal instability by formulation and process optimization, Eur. J. Pharm. Biopharm 165 (2021) 345–360, 10.1016/j.ejpb.2021.05.024. 34052428

[63] Jacob S, Kather FS, Boddu SHS, Attimarad M, Nair AB, Nanosuspension innovations: expanding Horizons in drug delivery techniques, Pharmaceutics 17 (1) (2025) 136, 10.3390/pharmaceutics17010136. 39861782 PMC11768797

[64] Abou-Saleh RH, Delaney A, Ingram N, Batchelor DVB, Johnson BRG, Charalambous A, Bushby RJ, Peyman SA, Coletta PL, Markham AF, Evans SD, Freeze-dried therapeutic microbubbles: stability and gas exchange, ACS Appl. Bio Mater 3 (11) (2020) 7840–7848, 10.1021/acsabm.0c00982. 35019524

[65] Far J, Abdel-Haq M, Gruber M, Abu Ammar A, Developing biodegradable nanoparticles loaded with mometasone furoate for potential nasal drug delivery, ACS Omega 5 (13) (2020) 7432–7439, 10.1021/acsomega.0c00111. 32280885 PMC7144157

[66] Andreana I, Bincoletto V, Manzoli M, Rodà F, Giarraputo V, Milla P, Arpicco S, Stella B, Freeze drying of polymer nanoparticles and liposomes exploiting different saccharide-based approaches, Materials 16 (3) (2023) 1212, 10.3390/ma16031212. 36770218 PMC9921637

[67] Amin K, Dannenfelser R, Zielinski J, Wang B, Lyophilization of polyethylene glycol mixtures, J Pharm Sci 93 (9) (2004) 2244–2249, 10.1002/jps.20135. 15295785

[68] Luo W-C, O’Reilly Beringhs A, Kim R, Zhang W, Patel SM, Bogner RH, Lu X, Impact of formulation on the quality and stability of freeze-dried nanoparticles, Eur. J. Pharm. Biopharm 169 (2021) 256–267, 10.1016/j.ejpb.2021.10.014. 34732383

[69] Jakubowska E, Bielejewski M, Milanowski B, Lulek J, Freeze-drying of drug Nanosuspension– study of formulation and processing factors for the optimization and characterization of redispersible cilostazol nanocrystals, J. Drug Deliv. Sci. Technol 74 (2022) 103528, 10.1016/j.jddst.2022.103528.

[70] Weisman RB, Fluorimetric characterization of single-walled carbon nanotubes, Anal. Bioanal. Chem 396 (3) (2010) 1015–1023, 10.1007/s00216-009-3062-8. 19763551

[71] Parnsubsakul A, Sapcharoenkun C, Warin C, Ekgasit S, Pienpinijtham P, Selection of cryoprotectants for freezing and freeze-drying of gold nanoparticles towards further uses in various applications, Colloids Surf. B Biointerfaces 217 (2022) 112702, 10.1016/j.colsurfb.2022.112702. 35863234

[72] Assembly of Poly(Vinyl alcohol) and DNA via hydrogen bonds induced by high hydrostatic pressurization, Sensor. Mater 607 (2014), 10.18494/SAM.2014.1023.

[73] Nadeem A, Kindopp A, Wyllie I, Hubert L, Joubert J, Lucente S, Randall E, Jena PV, Roxbury D, Enhancing intracellular optical performance and stability of engineered nanomaterials via aqueous two-phase purification, Nano Lett. 23 (14) (2023) 6588–6595, 10.1021/acs.nanolett.3c01727. 37410951 PMC11068083

[74] Kotula AP, Snyder CR, Migler KB, Determining conformational order and crystallinity in polycaprolactone via raman spectroscopy, Polymer (Guildf.) 117 (2017) 1–10, 10.1016/j.polymer.2017.04.006. 28824207 PMC5557303

[75] McCreery RL, Raman Spectroscopy for Chemical Analysis, John Wiley & Sons, Inc., 2000.

[76] Wang B, Gupta AK, Huang J, Vedala H, Hao Q, Crespi VH, Choi W, Eklund PC, Effect of bending on single-walled carbon nanotubes: a raman scattering study, Phys. Rev. B 81 (11) (2010) 115422, 10.1103/PhysRevB.81.115422.

[77] López-Lorente AI, Simonet BM, Valcárcel M, Raman spectroscopic characterization of single walled carbon nanotubes: influence of the sample aggregation state, Analyst 139 (1) (2014) 290–298, 10.1039/C3AN00642E. 24255912

[78] Fonte P, Soares S, Costa A, Andrade JC, Seabra V, Reis S, Sarmento B, Effect of cryoprotectants on the porosity and stability of insulin-loaded PLGA nanoparticles after freeze-drying, Biomatter 2 (4) (2012) 329–339, 10.4161/biom.23246. 23507897 PMC3568117

[79] Lee J, Cheng Y, Critical freezing rate in freeze drying nanocrystal dispersions, J. Contr. Release 111 (1–2) (2006) 185–192, 10.1016/j.jconrel.2005.12.003. 16430987

[80] Wolkers WF, Oliver AE, Tablin F, Crowe JH, A fourier-transform infrared spectroscopy study of sugar glasses, Carbohydr. Res 339 (6) (2004) 1077–1085, 10.1016/j.carres.2004.01.016. 15063194

[81] Le Losq C, Cody GD, Mysen BO, Complex IR spectra of OH- groups in silicate glasses: implications for the use of the 4500 Cm-1 IR peak as a marker of OH-groups concentration, Am. Mineral 100 (4) (2015) 945–950, 10.2138/am-2015-5076.

[82] Brogly M, Bistac S, Bindel D, Adsorption and structuration of PEG thin films: influence of the substrate chemistry, Polymers 16 (9) (2024) 1244, 10.3390/polym16091244. 38732713 PMC11085855

[83] Fonte P, Soares S, Sousa F, Costa A, Seabra V, Reis S, Sarmento B, Stability study perspective of the effect of freeze-drying using cryoprotectants on the structure of insulin loaded into PLGA nanoparticles, Biomacromolecules 15 (10) (2014) 3753–3765, 10.1021/bm5010383. 25180545

[84] Patel M, Park JK, Jeong B, Rediscovery of Poly(Ethylene Glycol)s as a cryoprotectant for mesenchymal stem cells, Biomater. Res 27 (1) (2023), 10.1186/s40824-023-00356-z. PMC9942331 36803669

[85] Thakral S, Sonje J, Munjal B, Bhatnagar B, Suryanarayanan R, Mannitol as an excipient for lyophilized injectable formulations, J Pharm Sci 112 (1) (2023) 19–35, 10.1016/j.xphs.2022.08.029. 36030846

[86] Crowe JH, Carpenter JF, Crowe LM, The role of vitrification in anhydrobiosis, Annu. Rev. Physiol 60 (1) (1998) 73–103, 10.1146/annurev.physiol.60.1.73. 9558455

[87] Wang G, Yu X, Lu Z, Yang Y, Xia Y, Lai PF-H, Ai L, Optimal combination of multiple cryoprotectants and freezing-thawing conditions for high lactobacilli survival rate during freezing and frozen storage, LWT 99 (2019) 217–223, 10.1016/j.lwt.2018.09.065.

[88] Feng B, Frykholm K, Nordén B, Westerlund F, DNA strand exchange catalyzed by molecular crowding in PEG solutions, Chem. Commun 46 (43) (2010) 8231, 10.1039/c0cc03117h. 20877908

[89] Collette D, Dunlap D, Finzi L, Macromolecular crowding and DNA: bridging the gap between in vitro and in vivo, Int. J. Mol. Sci 24 (24) (2023) 17502, 10.3390/ijms242417502. 38139331 PMC10744201

[90] Nii D, Hayashida T, Umemura K, Controlling the adsorption and desorption of double-stranded DNA on functionalized carbon nanotube surface, Colloids Surf. B Biointerfaces 106 (2013) 234–239, 10.1016/j.colsurfb.2013.01.054. 23434717

[91] Fagan JA, Aqueous two-polymer phase extraction of single-wall carbon nanotubes using surfactants, Nanoscale Adv. 1 (9) (2019) 3307–3324, 10.1039/C9NA00280D. 36133572 PMC9417344

[92] Marenduzzo D, Finan K, Cook PR, The depletion attraction: an underappreciated force driving cellular organization, J. Cell Biol 175 (5) (2006) 681–686, 10.1083/jcb.200609066. 17145959 PMC2064666

[93] Tian Y, Sun D-W, Xu L, Fan T-H, Zhang S-T, Zhu Z, Bioinspired cryoprotectants enabled by binary natural deep eutectic solvents for sustainable and green cryopreservation, ACS Sustain Chem Eng 10 (23) (2022) 7677–7691, 10.1021/acssuschemeng.2c01578.

[94] Mitchell DE, Fayter AER, Deller RC, Hasan M, Gutierrez-Marcos J, Gibson MI, Ice-recrystallization inhibiting polymers protect proteins against freeze-stress and enable glycerol-free cryostorage, Mater. Horiz 6 (2) (2019) 364–368, 10.1039/C8MH00727F. 30931129 PMC6394881

[95] Murray KA, Gibson MI, Chemical approaches to cryopreservation, Nat. Rev. Chem 6 (8) (2022) 579–593, 10.1038/s41570-022-00407-4. PMC9294745 35875681

[96] Maji D, Maity NC, Biswas R, Structure and dynamics of a glucose-based cryoprotectant mixture: a computer simulation study, Theor. Chem. Acc 142 (5) (2023) 43, 10.1007/s00214-023-02986-x.

[97] Yu G, Li R, Hubel A, Interfacial interactions of sucrose during cryopreservation detected by Raman spectroscopy, Langmuir 35 (23) (2019) 7388–7395, 10.1021/acs.langmuir.8b01616. 30398347 PMC8023323

[98] Pardeshi SR, Deshmukh NS, Telange DR, Nangare SN, Sonar YY, Lakade SH, Harde MT, Pardeshi CV, Gholap A, Deshmukh PK, More MP, Process development and quality attributes for the freeze-drying process in pharmaceuticals, biopharmaceuticals and nanomedicine delivery: a state-of-the-art review, Futur J Pharm Sci 9 (1) (2023) 99, 10.1186/s43094-023-00551-8.

[99] Benoit M, Desnues B, Mege J-L, Macrophage polarization in bacterial infections, J. Immunol 181 (6) (2008) 3733–3739, 10.4049/jimmunol.181.6.3733. 18768823

[100] Gravely M, Kindopp A, Hubert L, Card M, Nadeem A, Miller C, Roxbury D, Aggregation reduces subcellular localization and cytotoxicity of single-walled carbon nanotubes, ACS Appl. Mater. Interfaces 14 (17) (2022) 19168–19177, 10.1021/acsami.2c02238. 35438957 PMC11068084

[101] Venter G, Oerlemans FTJJ, Wijers M, Willemse M, Fransen JAM, Wieringa B, Glucose controls morphodynamics of LPS-stimulated macrophages, PLoS One 9 (5) (2014) e96786, 10.1371/journal.pone.0096786. 24796786 PMC4010488

[102] Pavlou S, Wang L, Xu H, Chen M, Higher phagocytic activity of thioglycollate-elicited peritoneal macrophages is related to metabolic status of the cells, J. Inflamm 14 (1) (2017) 4, 10.1186/s12950-017-0151-x. PMC5301433 28203119

[103] Friedl JD, Nele V, De Rosa G, Bernkop-Schnürch A Bioinert, Stealth or interactive: how surface chemistry of nanocarriers determines their fate in vivo, Adv. Funct. Mater 31 (34) (2021).

