# Supplementary material for: Enhancing optical properties and stability of DNA-functionalized carbon nanotubes with cryoprotectant-mediated lyophilization
Source: Carbon N Y. Author manuscript; Available in PMC 2026 Feb 5. (PMC12872225; doi:10.1016/j.carbon.2025.121159)
Supplement: Supporting Information text [file NIHMS2140091-supplement-Supporting_Information_text.docx]

*Supporting Information for*

**Enhancing Optical Properties and Stability of DNA-Functionalized**

**Carbon Nanotubes with Cryoprotectant-Mediated Lyophilization**

*Aceer Nadeem^,1,2^, Aidan Kindopp^1^, Ella Junge^1^, Maryam Rahmani^1^, and Daniel Roxbury^1^**

^1^Department of Chemical Engineering, University of Rhode Island,

Kingston, Rhode Island 02881, United States

^2^School of Chemistry and Biochemistry, Georgia Institute of Technology,

Atlanta, Georgia 30322, United States

*Corresponding Author Email: roxbury@uri.edu

**Table S1.** List of cryoprotectants investigated in the study with their respective molecular weights and weight percentages used in all experiments

| **Cryoprotectant** | **Molecular Weight / Weight Percent** |
| --- | --- |
| Polyethylene glycol | MW – 1500 / 0.25 wt.% |
| Polyvinyl alcohol | MW – 10,000 / 0.25 wt.% |
| Glucose | 10 wt.% |
| Sucrose | 10 wt.% |
| Mannitol | 10 wt.% |

**Table S2.** Evaluated parameters during the final selection of glucose and polyethylene glycol as cryoprotectants for process of SWCNT lyophilization.

| **Parameter** |
| --- |
| Re-dispersibility |
| Long term stability – time* |
| Long term stability – storage temp* |
| Degree of aggregation |
| Degree of crystallinity |
| Change in NIR-fluorescence signal |
| *In-vitro* signal stability |

**Table S3.** Detailed table describing absorbance change observed at 990nm in SWCNT (L+R) samples with respect to the as dispersed SWCNT sample.

| **CP Sample** | **Absorbance change (a.u.)** | **Trend vs. Control** |
| --- | --- | --- |
| PEG | -0.0351 | Decrease |
| PVA | -0.1246 | Large Decrease |
| Glucose | 0.0324 | Increase |
| Sucrose | 0.0501 | Increase |
| Mannitol | 0.034 | Increase |

**Table S4.** Near-Infrared fluorescence peak intensities for all samples, including lyophilized samples with different cryoprotectants and as-dispersed SWCNT control sample. Intensities are shown for all major fluorescence bands identified in samples analyzed during lyophilization experiments.

| **Band #** | **λ Range** | **GT_30_ SWCNTs** | **Glucose** | **Mannitol** | **PEG** | **PVA** | **Sucrose** |
| --- | --- | --- | --- | --- | --- | --- | --- |
| Band 1 | 1020-1080 | 1179.12 | 1061.25 | 574.47 | 934.21 | 998.55 | 1069.74 |
| Band 2 | 1081-1140 | 2192.36 | 2217.91 | 1093.22 | 1961.44 | 1585.8 | 2223.68 |
| Band 3 | 1141-1250 | 1473.38 | 1310.35 | 627.15 | 1537.38 | 1382.7 | 1336.96 |
| Band 4 | 1251-1350 | 670.39 | 696.57 | 308.86 | 746.51 | 704.2 | 727.14 |


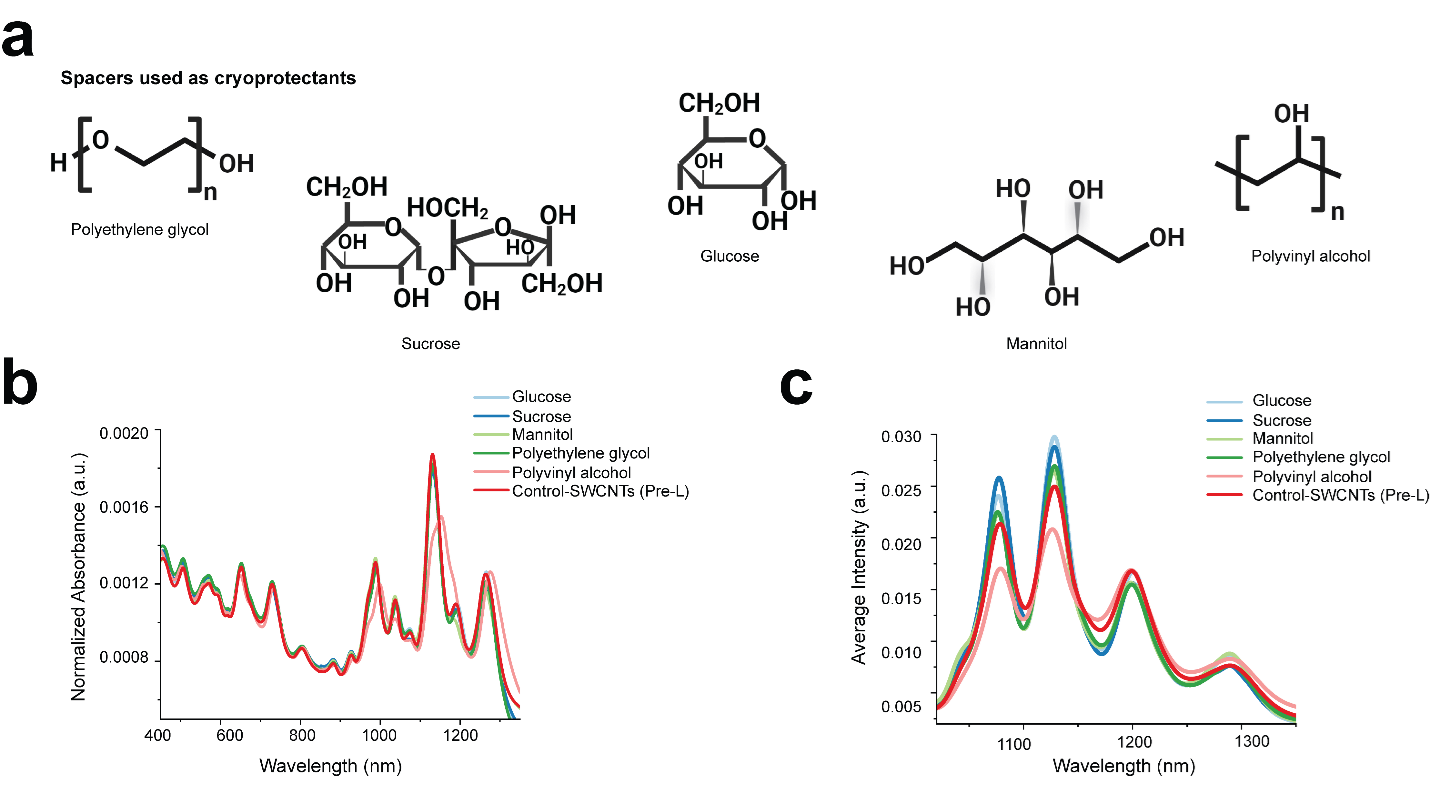


**Figure S1. Cryoprotectants for lyophilization.** Schematics showing chemical structures of cryoprotectants (CPs) that were investigated in this study.


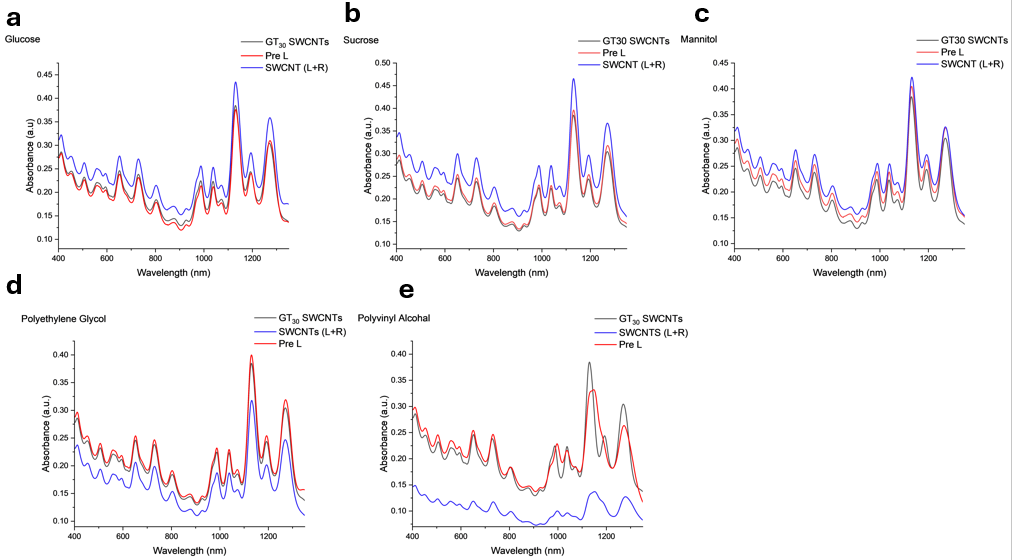


**Figure S2. Absorbance characterization of lyophilized DNA-SWCNTs.** Absorbance spectroscopy plots comparing spectrum of SWCNT samples with all CPs investigated pre-lyophilization (Pre L) and reconstituted lyophilized SWCNTs (L+R) for (a) glucose, (b) sucrose, (c) mannitol, (d) polyethylene glycol, and (e) polyvinyl alcohol.


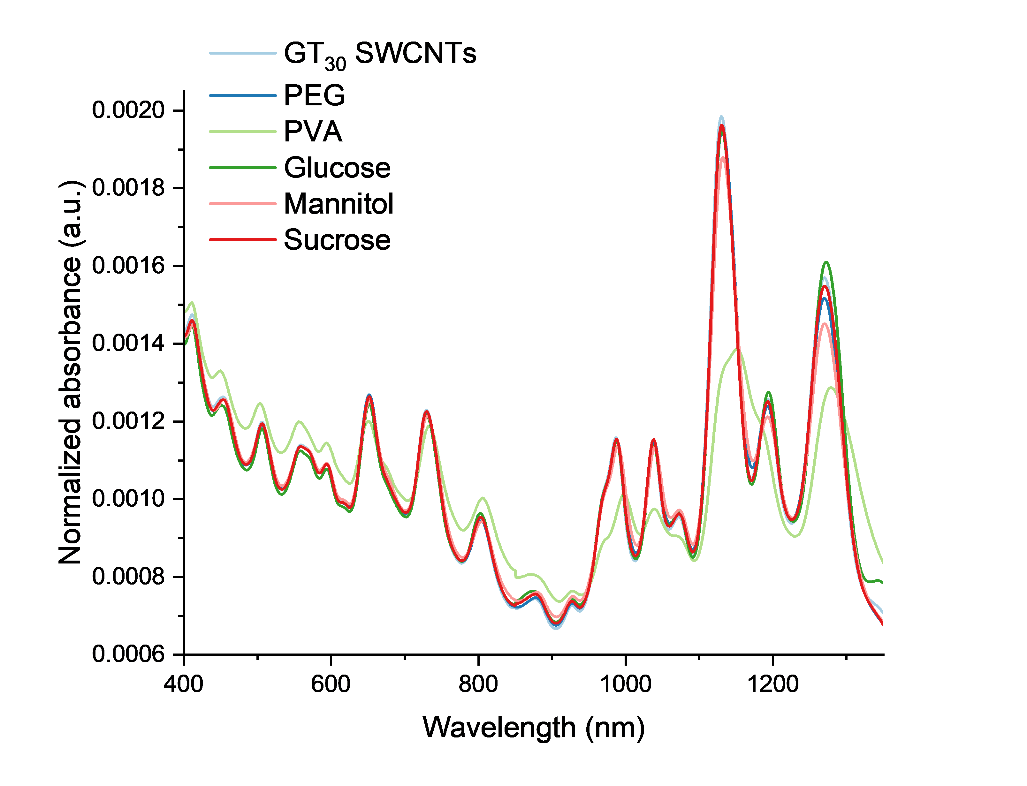


**Figure S3.** Normalized absorbance of all SWCNT (L+R) samples and the as-dispersed GT_30_ SWCNT sample.

**
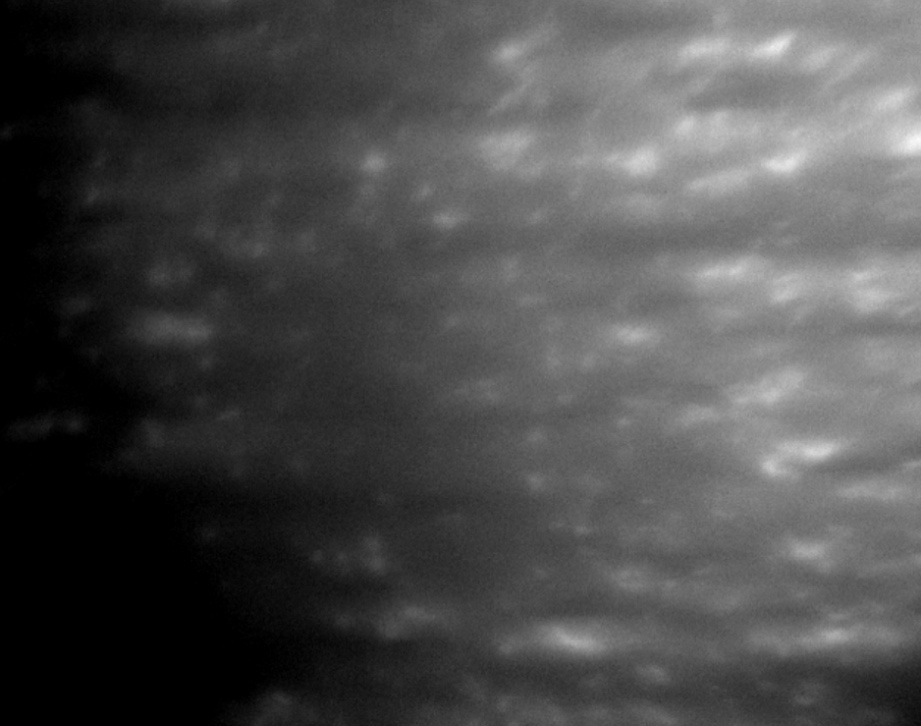
**

**Figure S4.** NIR fluorescence image showing small bundling in DNA-SWCNT L+R Mannitol sample (Size of scale bar is 15µm.)


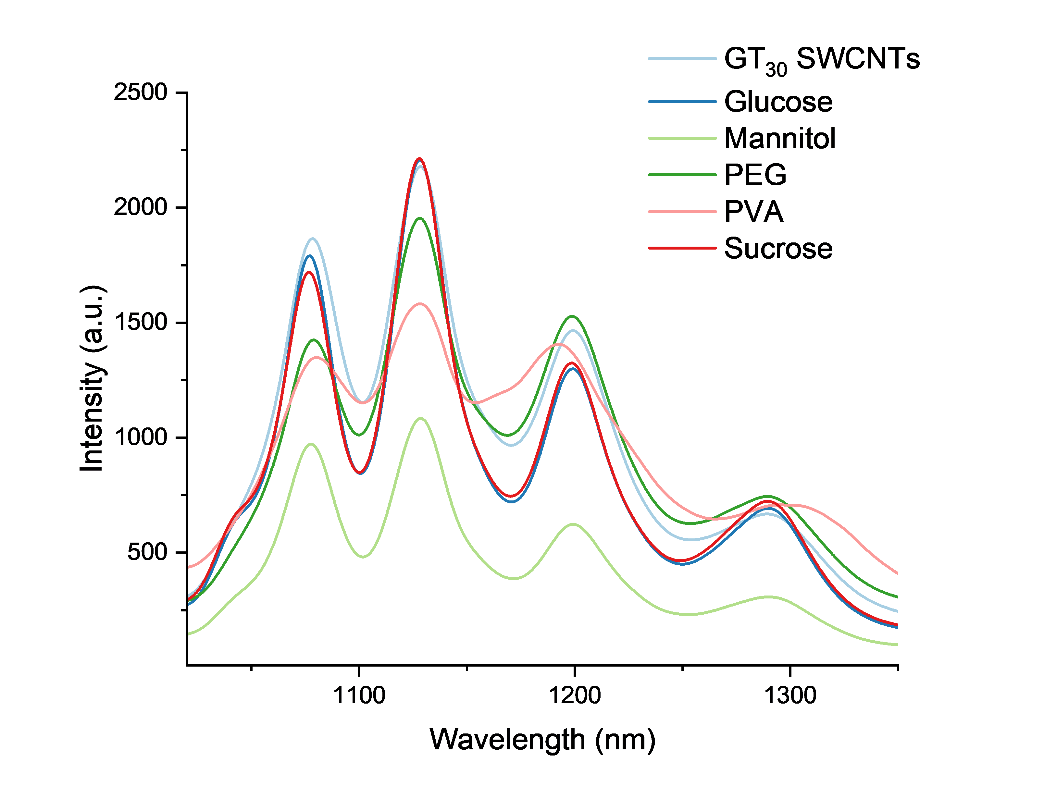


**Figure S5.** NIR fluorescence plot of DNA-SWCNT samples with all CPs investigated upon reconstitution after lyophilization and compared to as dispersed SWCNTs as absolute intensity


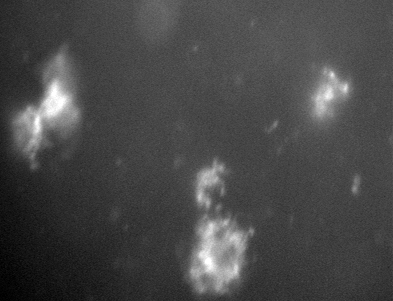


**Figure S6**. NIR broadband fluorescence image (900-1400 nm) showing substantial aggregation in DNA-SWCNT L+R PVA sample (Size of scale bar is 15µm.)


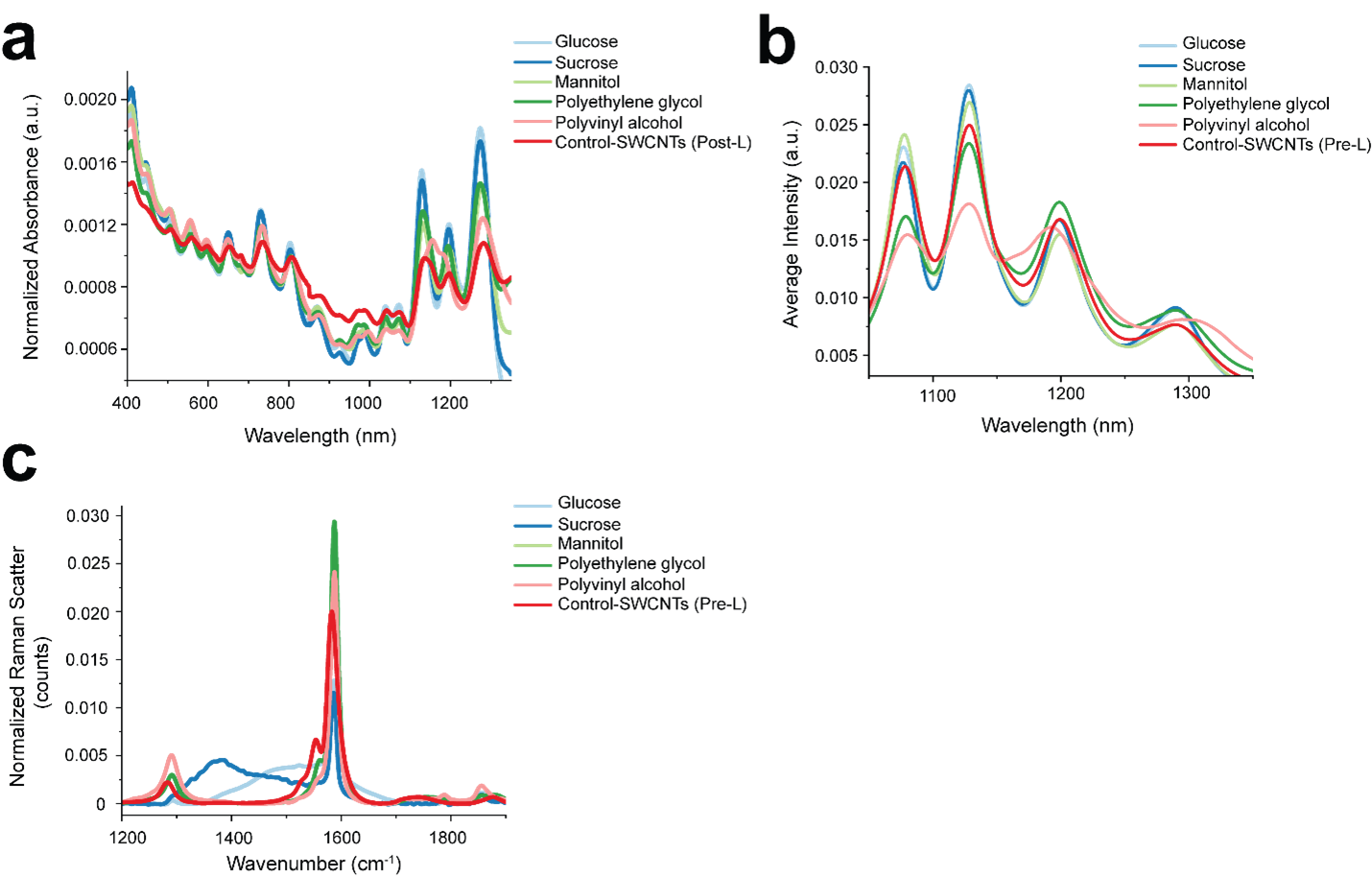


**Figure S7. Raman characterization of lyophilized DNA-SWCNTs.** Raman scatter spectra showing comparison between lyophilized samples and the control DNA-SWCNT sample (as produced, non-lyophilized sample).


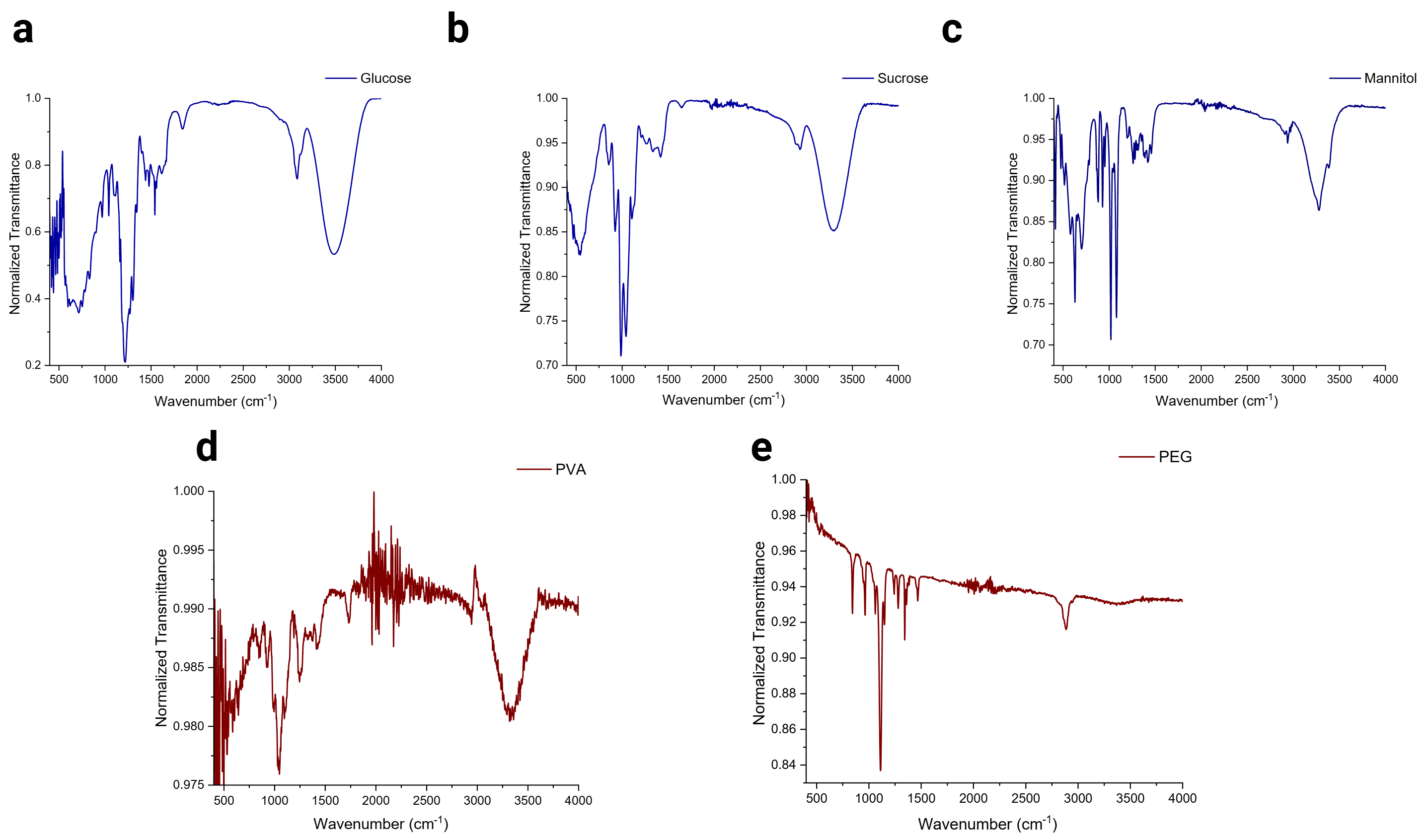


**Figure S8.** Fourier Transform Infrared Spectroscopy (FTIR) comparison of all lyophilized DNA-SWCNTs powders.


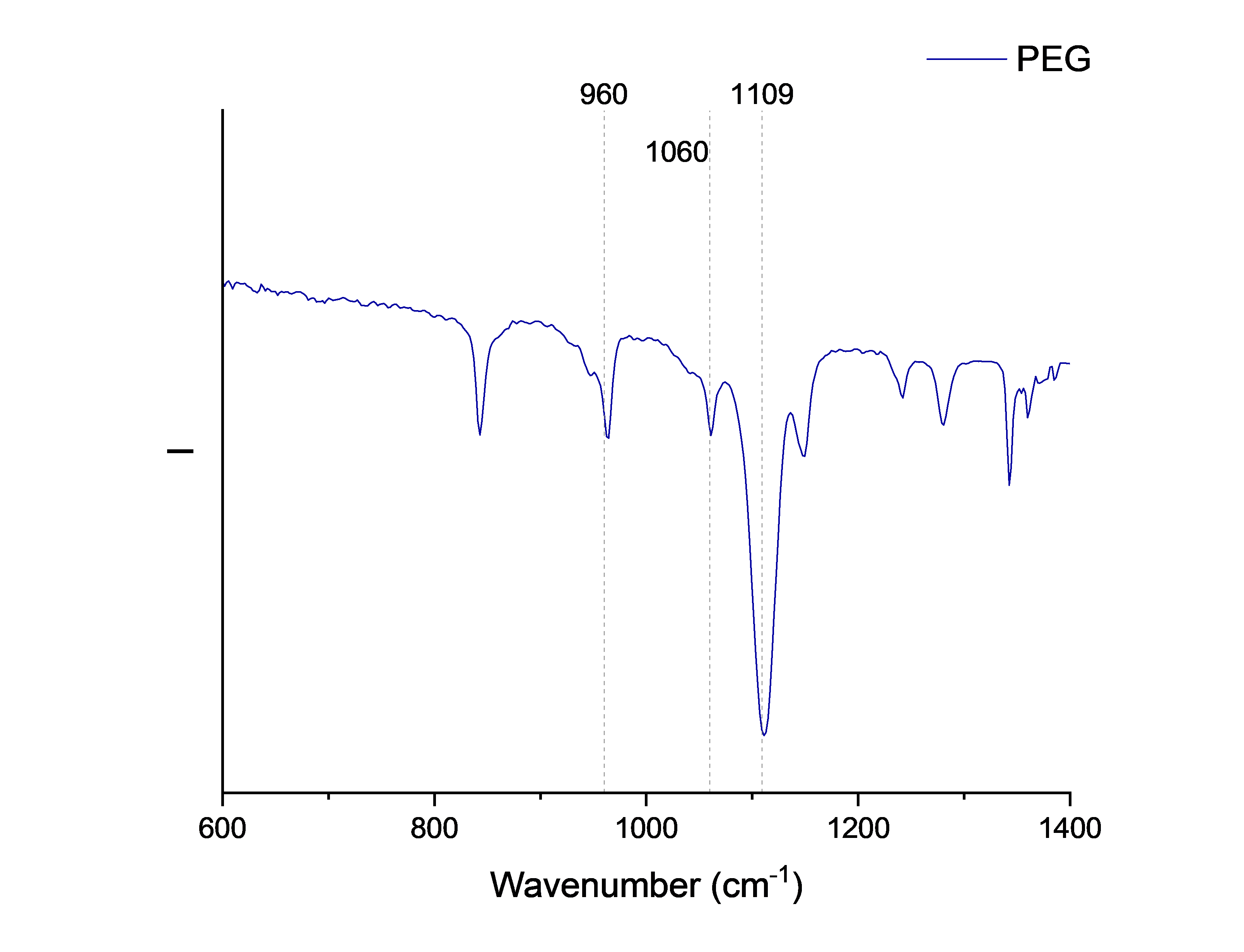


Transmittance

**Figure S9**. Fourier Transform Infrared Spectroscopy (FTIR) of all lyophilized DNA-SWCNTs with PEG showing fingerprint peaks.

Explanation for Amorphous and Crystaline structure:

FTIR was performed for qualitative analysis of peaks to account for structural information. All sugars have broadened O-H peaks between 3000-3500 cm^-1^. This broadening is primarily due to the presence of an amorphous structure. For glucose fingerprint peaks between 500 and 1500 cm^-1^ also share a degree of broadening (are not very sharp) indicative of an amorphous structure. Comparatively, peaks in Sucrose and Mannitol are sharper in nature as compared to other sugar cryoprotectant additives, indicating a mixed structure of both amorphous and crystalline nature. For polymer cryoprotectant additives, PVA was observed to have a more amorphous structure due to peak broadening at the O-H peaks (3000-3500) as well as its fingerprint at 1000-1500cm^-1^. In contrast, PEG shows sharp characteristic fingerprint peaks at 960, 1060, and 1109 cm^-1^ indicative of a crystalline structure.


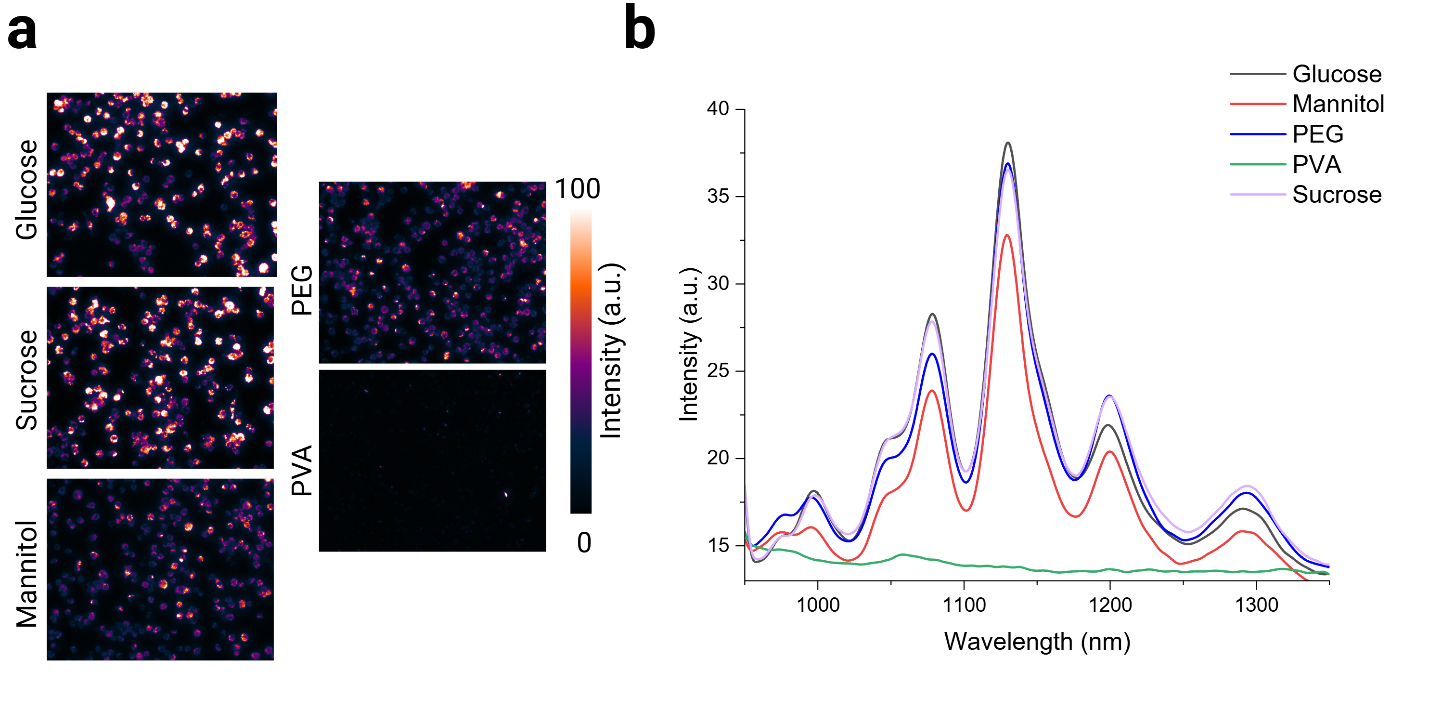


**Figure S10. *In vitro* cell investigations of DNA-SWCNT L+R samples.** (a) NIR broadband fluorescence (900-1400 nm) images of all DNA-SWCNT L+R samples incubated with murine macrophages. Images have been globally contrasted to the brightest image. Scale bar =.15 µm (b) Intracellular NIR fluorescence spectrum for all conditions as shown in (a).

Figure S10 shows NIR broadband fluorescence images (i.e. integrated fluorescence intensity from 900-1400 nm) and spectral response from all DNA-SWCNT L+R samples acquired at the 1-hour timepoint. As expected, glucose, sucrose, and PEG samples performed similarly in response and displayed good NIR fluorescence intensity signals. The mannitol sample experienced a decreased intensity as compared to the other CPs. Finally, the PVA sample displayed limited internalization into cells, with most DNA-SWCNTs becoming aggregated and either getting removed in the washing step or getting stuck to the cell membrane as depicted in Figure S11b. Spectral analyses of such samples were disregarded as we chose to focus only on DNA-SWCNTs that internalized into the cells and gave the best results.


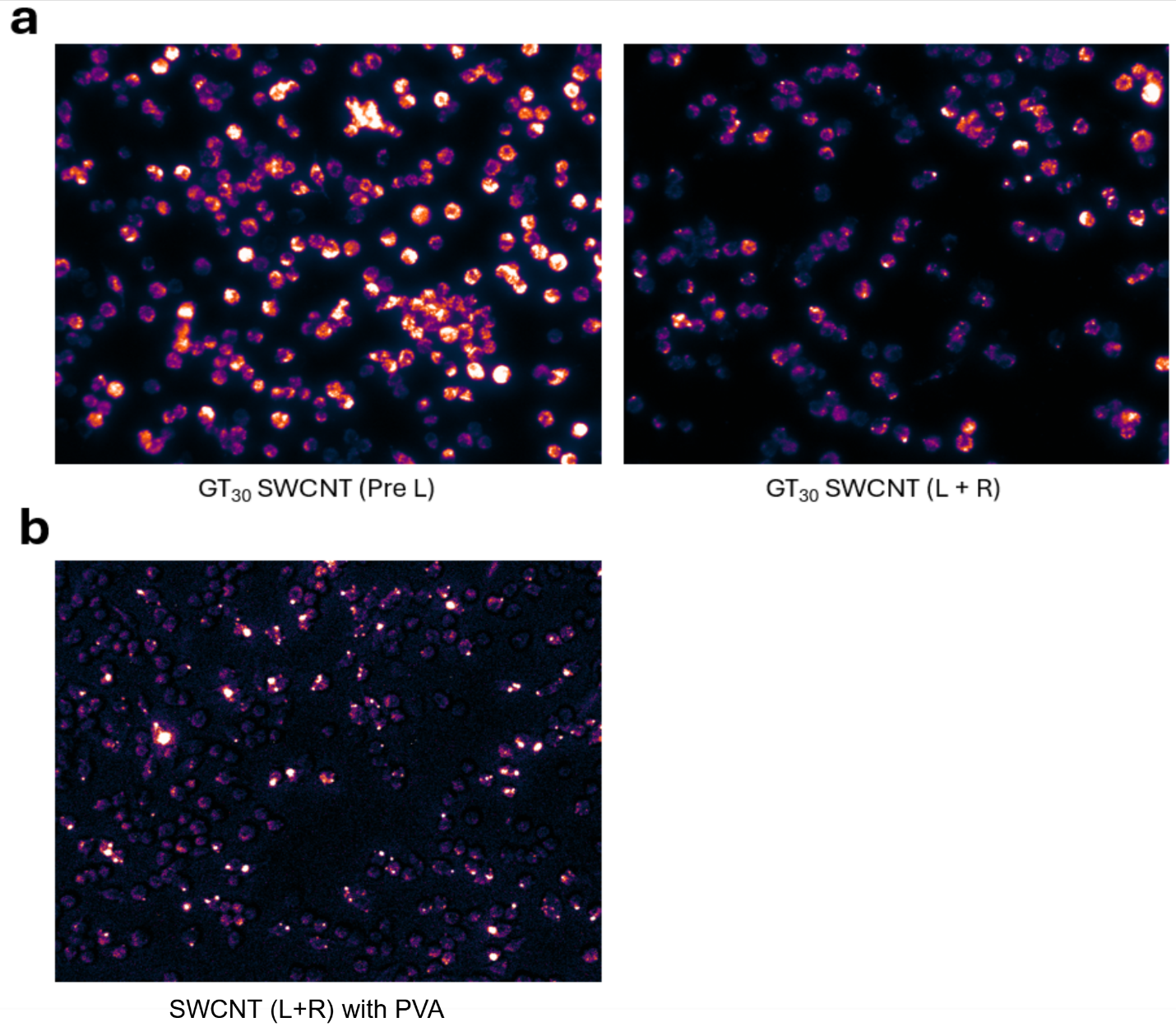


**Figure S11.** Intracellular NIR fluorescence broadband images of (a) GT_30_ SWCNT sample before (as dispersed) and after L+R without the addition of any CPs. (b) Visible aggregation of DNA-SWCNT L+R sample with PVA. Image was contrasted to show aggregation and cells. (Size of scale bar is 15µm.)


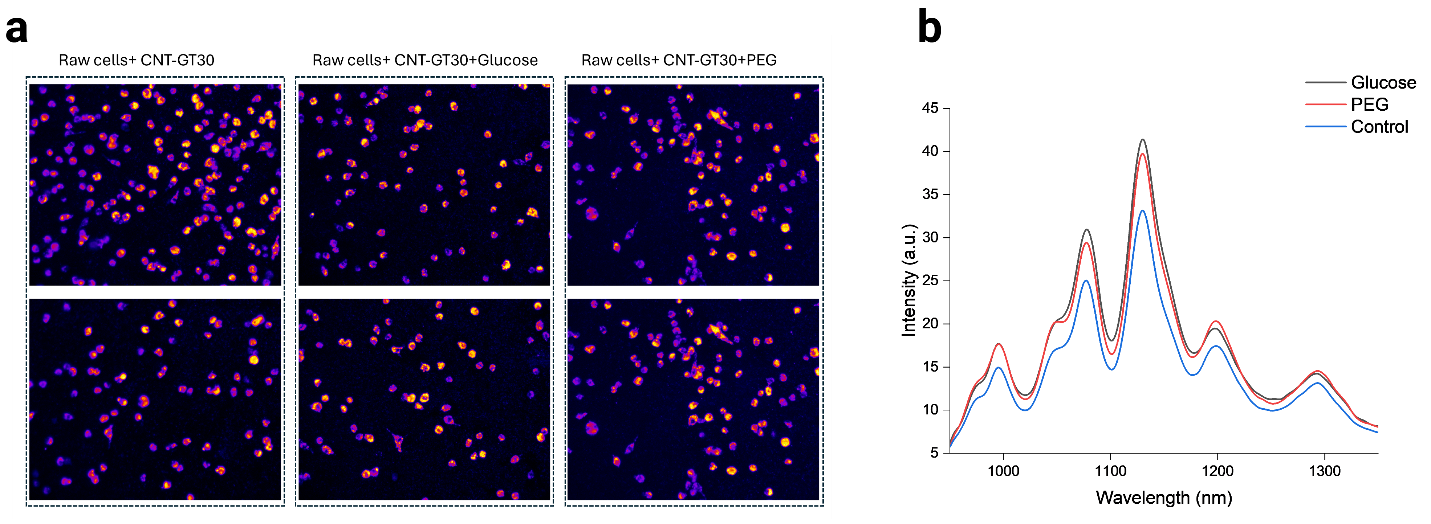


**Figure S12.**  **Further *In vitro* cell investigations to observe enhanced optical response of glucose and PEG additives to SWCNTs.** (a) NIR broadband fluorescence (900-1400 nm) images of all DNA-SWCNT incubated with murine macrophages in presence of PEG and glucose. Images have been globally contrasted to the brightest image. Scale bar =15 µm (b) Intracellular NIR fluorescence spectrum for all conditions as shown in (a).


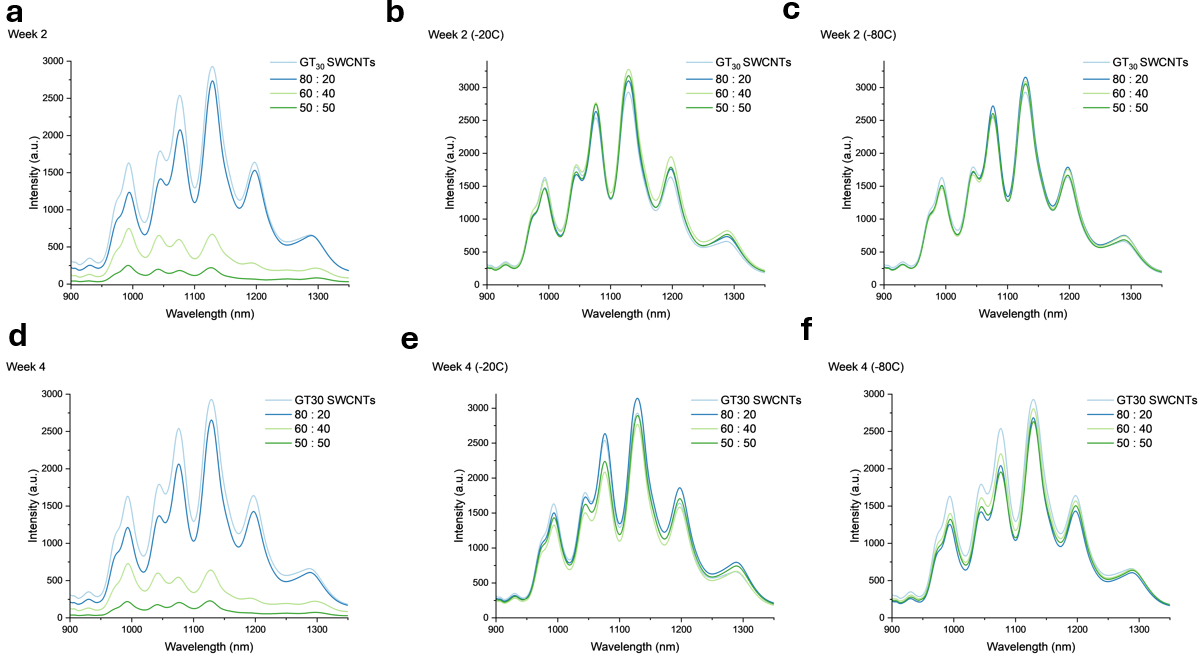


**Figure S13. Long term stability investigation.** Investigations into the long-term stability of chosen glucose:PEG ratios for 2 weeks at (a) room temperature, (b) -20°C, or (c) -80°C and for 4 weeks at (c) room temperature, (d) -20°C, or (e) -80°C.

**
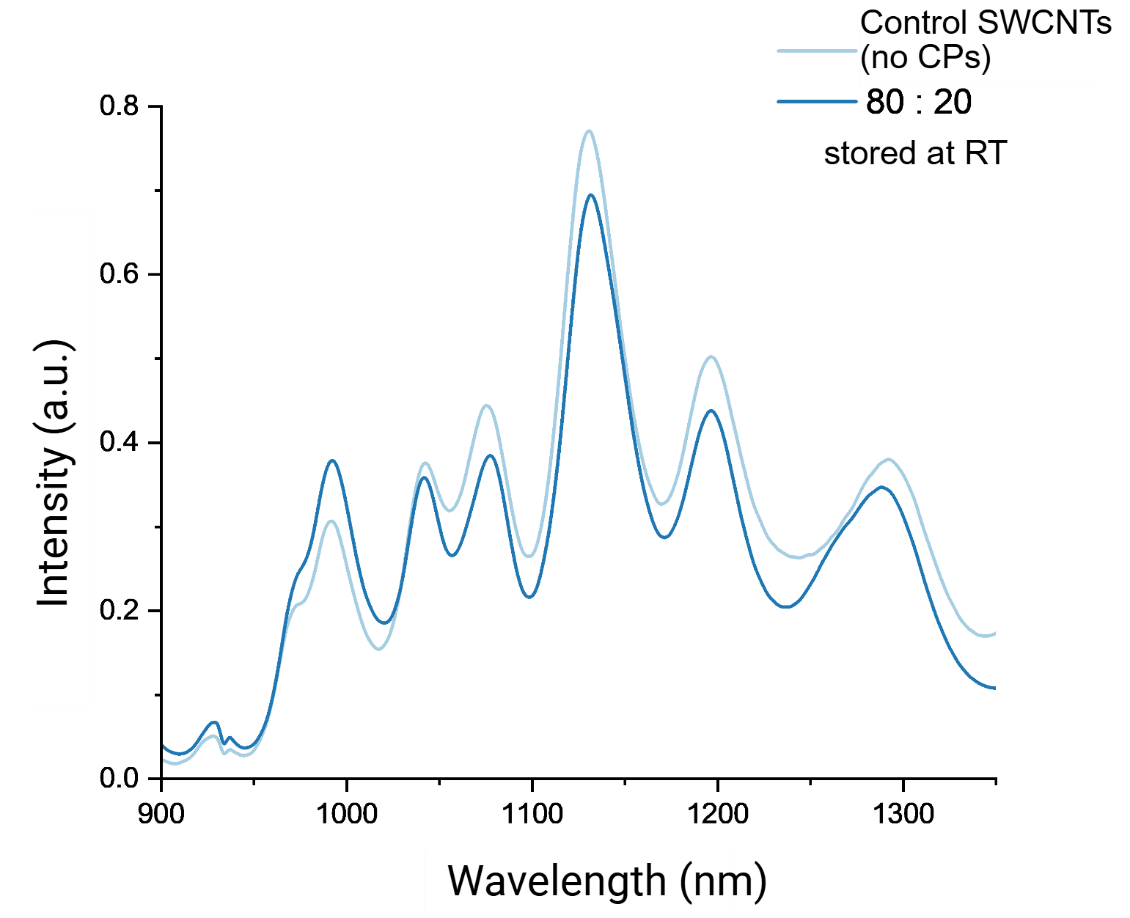
**

**Figure S14.** Near infrared fluorescence response comparison between control DNA-SWCNT (as dispersed) sample and the 80:20 glucose:PEG ratio L+R sample stored at room temperature for 1 year.

**
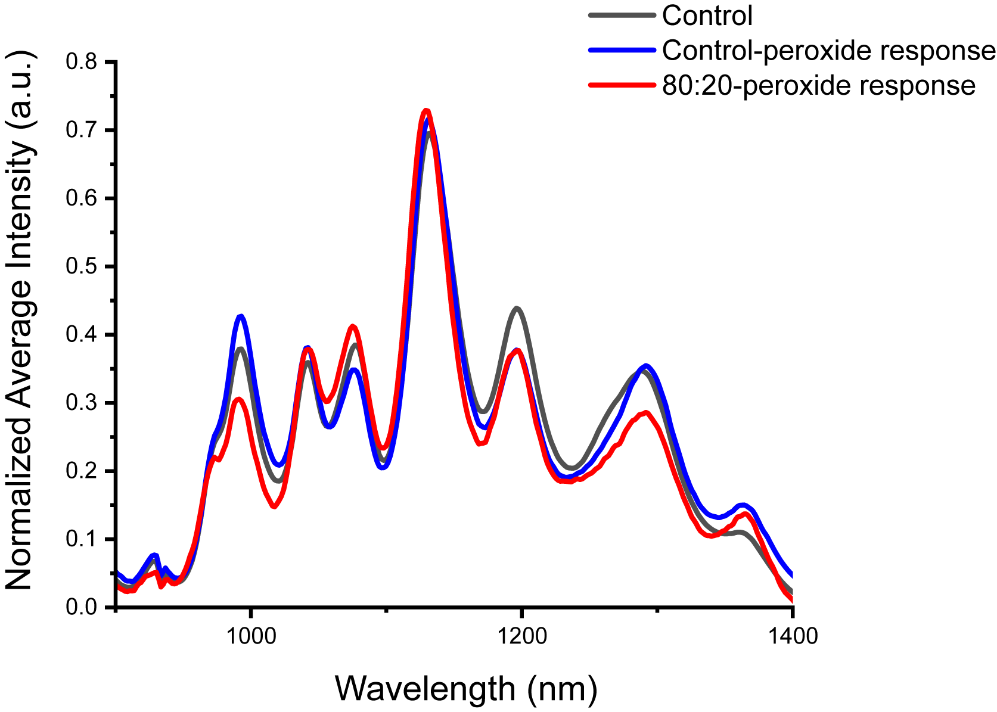
**

**Figure S15.** Near infrared fluorescence response comparison between control DNA-SWCNT (as dispersed) sample and the 80:20 glucose:PEG ratio L+R sample upon the addition of 2 mM hydrogen peroxide.

**
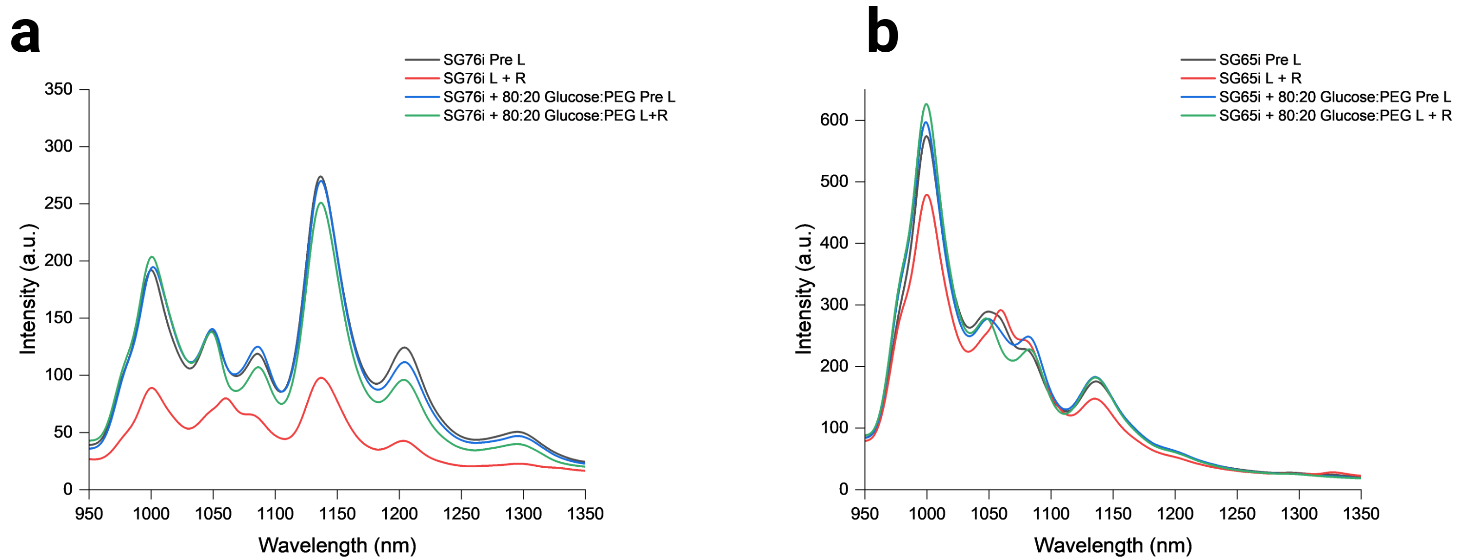
**

**Figure S16.** Near infrared fluorescence response comparison between control DNA-SWCNT (as dispersed) sample and the 80:20 glucose:PEG ratio samples both Pre L and L+R (a) for SG76i and (b) SG65i.
